# Sedentary Life and Reduced Mastication Impair Spatial Learning and Memory and Differentially Affect Dentate Gyrus Astrocyte Subtypes in the Aged Mice

**DOI:** 10.3389/fnins.2021.632216

**Published:** 2021-04-15

**Authors:** Fabíola de Carvalho Chaves de Siqueira Mendes, Luisa Taynah Vasconcelos Barbosa Paixão, Daniel Guerreiro Diniz, Daniel Clive Anthony, Dora Brites, Cristovam Wanderley Picanço Diniz, Marcia Consentino Kronka Sosthenes

**Affiliations:** ^1^Laboratório de Investigações em Neurodegeneração e Infecção, Instituto de Ciências Biológicas, Hospital Universitário João de Barros Barreto, Universidade Federal do Pará, Belém, Brazil; ^2^Curso de Medicina, Centro Universitário do Estado do Pará, Belém, Brazil; ^3^Laboratório de Microscopia Eletrônica, Instituto Evandro Chagas, Belém, Brazil; ^4^Laboratory of Experimental Neuropathology, Department of Pharmacology, University of Oxford, Oxford, United Kingdom; ^5^Research Institute for Medicines (iMed.ULisboa), Faculty of Pharmacy, Universidade de Lisboa, Lisbon, Portugal; ^6^Department of Biochemistry and Human Biology, Faculty of Pharmacy, Universidade de Lisboa, Lisbon, Portugal

**Keywords:** masticatory activity, enriched environment, spatial learning and memory, astrocytes, morphometry, aging

## Abstract

To explore the impact of reduced mastication and a sedentary lifestyle on spatial learning and memory in the aged mice, as well as on the morphology of astrocytes in the molecular layer of dentate gyrus (MolDG), different masticatory regimens were imposed. Control mice received a pellet-type hard diet, while the reduced masticatory activity group received a pellet diet followed by a powdered diet, and the masticatory rehabilitation group received a pellet diet, followed by powder diet and then a pellet again. To mimic sedentary or active lifestyles, mice were housed in an impoverished environment of standard cages or in an enriched environment. The Morris Water Maze (MWM) test showed that masticatory-deprived group, regardless of environment, was not able to learn and remember the hidden platform location, but masticatory rehabilitation combined with enriched environment recovered such disabilities. Microscopic three-dimensional reconstructions of 1,800 glial fibrillary acidic protein (GFAP)-immunolabeled astrocytes from the external third of the MolDG were generated using a stereological systematic and random sampling approach. Hierarchical cluster analysis allowed the characterization into two main groups of astrocytes with greater and lower morphological complexities, respectively, AST1 and AST2. When compared to compared to the hard diet group subjected to impoverished environment, deprived animals maintained in the same environment for 6 months showed remarkable shrinkage of astrocyte branches. However, the long-term environmental enrichment (18-month-old) applied to the deprived group reversed the shrinkage effect, with significant increase in the morphological complexity of AST1 and AST2, when in an impoverished or enriched environment. During housing under enriched environment, complexity of branches of AST1 and AST2 was reduced by the powder diet (pellet followed by powder regimes) in young but not in old mice, where it was reversed by pellet diet (pellet followed by powder and pellet regime again). The same was not true for mice housed under impoverished environment. Interestingly, we were unable to find any correlation between MWM data and astrocyte morphological changes. Our findings indicate that both young and aged mice subjected to environmental enrichment, and under normal or rehabilitated masticatory activity, preserve spatial learning and memory. Nonetheless, data suggest that an impoverished environment and reduced mastication synergize to aggravate age-related cognitive decline; however, the association with morphological diversity of AST1 and AST2 at the MolDG requires further investigation.

## Highlights

- Reduction and rehabilitation of masticatory activity in mice influence spatial learning and memory.
- Environmental enrichment associated with rehabilitation of chewing activity in young and aging mice improves performance in the Morris Water Maze test in contrast to the impoverished environment.
- Aging and masticatory deprivation diversely affect AST1 and AST2 morphological complexities at the molecular layer of dentate gyrus.

## Introduction

The removal of molar teeth has been associated with impaired spatial learning in middle age and aggravation as aging progresses, suggesting that decreased masticatory activity (chewing) accelerates cognitive decline in aging (Watanabe et al., 2001b; Kondo et al., 2016; Dintica et al., 2020), while its preservation is important for cognitive function (Teixeira et al., 2014; Chen et al., 2015). In association with the deleterious effect on spatial learning and memory, elderly mice with occlusal disharmony also exhibit cognitive decline that is associated with amyloid-beta production in the hippocampus (Ekuni et al., 2013) and reduced neuronal density in the CA3 region (Kubo et al., 2007). Likewise, a reduction in masticatory activity diminishes neurogenesis and the expression of neurotrophic factor brain-derived neurotrophic factor (BDNF) receptors in the dentate gyrus, as well as in the CA1 and CA3 regions of the hippocampus (Fukushima-Nakayama et al., 2017). Thus, abnormal masticatory activity impairs morphofunctional features of hippocampus with synaptic dysfunctional activity, suggesting that all life healthy chewing is essential for hippocampal-dependent cognitive function (Piancino et al., 2020).

A possible link between masticatory dysfunction and the activity of hypothalamic–hypophysis–adrenal (H–H–A) axis (Azuma et al., 2017) has been demonstrated, that induces a continuous increase in circulating levels of glucocorticoids, interruption of the negative feedback system, ultimately leading to chronic neurotoxicity (Ono et al., 2010; Chen et al., 2015; Kubo et al., 2015). By contrast, chewing also seems to be part of a stress-coping behavior, as mastication during stress suppresses stress-induced increases in plasma corticosterone and catecholamines (Kubo et al., 2015).

Thus, considering that a reduction in masticatory activity induces negative effects on brain function and that its stimulus may have the opposite effect, we tested whether maintenance or rehabilitation acts on delaying or recovering effects of pathological or senile cognitive decline.

Two other important conditions that are known to contribute to increase aging-associated cognitive decline in both humans and experimental models are the sedentary lifestyle (Leon and Woo, 2018; Cunningham et al., 2020) and the chronic low-grade neuroinflammation of the aged brain (Franceschi et al., 2007; Olmedillas Del Moral et al., 2019). The heterogeneity in the expression profile of proteins involved in astrocyte function and the loss of functional properties may predict the selective vulnerability of brain regions to specific diseases, as well as to age-related cognitive decline (Brites, 2015; Matias et al., 2019; Valles et al., 2019). The influence of these conditions on the cognitive decline late in life is dependent on personal genetics (Lupo et al., 2019; Kauppi et al., 2020), repetitive inflammatory stimuli (Valles et al., 2019), and environmental exposures (Guarente, 2014; Alsegiani and Shah, 2020; Queen et al., 2020).

A recent review gathered evidence on the existence of the direct association between total sedentary time and lower global cognition in elderly people without dementia, thus requiring further studies to clarify what kind of sedentary behavior associates with age-related cognitive decline (Maasakkers et al., 2020). Exercise, cognitively stimulating activities, nutrition, and social connection ultimately will allow elderly individuals to adjust their lifestyle and keep their brain healthy (Mintzer et al., 2019). Since neuroprotective actions require a plastic neural substrate to settle, and the experimental enriched environment may relate to neuroplasticity, we have compared animals raised in an enriched environment with those kept in an impoverished environment one. Environmental enrichment for rodents promotes multiple levels of somatosensory, motor, visuospatial, and cognitive stimulation (van Praag, 2008). Actually, environmental enrichment was shown to stimulate hippocampal neurogenesis and to promote better performances in learning and memory (Fares et al., 2013).

Recently, age-related cognitive decline was associated with molecular changes as well as with the dysregulation of the synaptic transcriptome in neurons and of the inflammatory transcriptome in glial cells (Lupo et al., 2019). Differences in transcriptomic analysis with age were found in the APP-PS1 mouse model of Alzheimer’s disease for microglia and astrocytes (Galatro et al., 2017; Olah et al., 2018; Angelova and Brown, 2019; Masuda et al., 2020; Tan et al., 2020). In the human brain, complex patterns of astrocyte-specific genes were also dependent on both regional identity and age, suggesting contrasting differences between the young and the aged brain (Soreq et al., 2017; Boisvert et al., 2018; Lupo et al., 2019). Age-related cognitive decline was found to be associated with the overexpression of astrocyte reactive markers and the elimination of synapses (Boisvert et al., 2018; Pan et al., 2020).

In addition to changes in gene expression, astrocytes also exhibit alterations in the morphology of their processes and cell soma in different areas of the brain [central nervous system (CNS)], which also differ among species (Oberheim et al., 2009; López-Hidalgo et al., 2016; Augusto-Oliveira et al., 2020; Klein et al., 2020; O’Leary et al., 2020). Age and environmental changes also influence the morphological appearance of astrocytes (Rodríguez et al., 2014; Palmer and Ousman, 2018; Verkhratsky et al., 2019; Klein et al., 2020). It is difficult to relate how the changes in astrocyte morphology associate with gene expression deregulation and cognitive decline, but it is now accepted that they are markers of degeneration and synaptic loss (Viola et al., 2009; Sampedro-Piquero et al., 2014; Diniz et al., 2016; Salois and Smith, 2016).

We have previously demonstrated that the reduction in masticatory activity by a powder diet on the aged mice previously fed with a hard diet led to spatial learning impairment in the Morris Water Maze (MWM) test (Mendes et al., 2013). However, a complete recovery of these deficits was produced by combining masticatory rehabilitation with long-term environmental enrichment, which may relate to astrocyte changes under the influence of age and environment. Therefore, we hypothesized that masticatory activity may interact with these variables and be differentially impact on astrocyte morphology.

The learning and spatial memory tested by MWM test (Morris, 1984) require the acquisition of spatial location of relevant extra- or intramaze visual cues that are subsequently processed and retrieved to successfully navigate toward the submerged platform and escape from water (Buccafusco, 2009). This hippocampal-dependent task (Morris et al., 1982; Eichenbaum, 1996; Ergorul and Eichenbaum, 2004) activates the perforant paths and stimulate the outer and middle thirds of mouse dentate gyrus molecular layer (van Groen et al., 2003), which are related to the circuits associated with object identity recognition and spatial memory, respectively (Hunsaker et al., 2007, 2008). Such findings determined the decision direct our studies to the outer third of the molecular layer of the dentate gyrus.

Thus, to investigate how environmental stimuli, aging, and masticatory activity could affect the dentate gyrus, we reconstructed the three-dimensional structure of astrocytes. We analyzed in detail their morphometry at the outer third of molecular layer given its relevance as an important route of afferents to the hippocampus, and we further quested these findings to the impact on learning and spatial memory of mice.

## Experimental Procedures

### Experimental Groups

All experimental procedures were submitted to the Ethics Committee in Research with Experimental Animals (CEPAE) of Biological Sciences Institute from Federal University of Pará [Universidade Federal do Pará (UFPA)] and were fully approved according to CEPAE-UFPA regulations (Report: 223-14). Six pups (females) from Swiss albino adult females (*Mus musculus*) on fifth postnatal day were selected from each litter and randomly organized. Mice remained with their mothers until the 21st postnatal day. At weaning, 132 females were organized in experimental groups, based on the three variables to be investigated: age, masticatory regime, and environment. All the mice from the different experimental groups were subjected to MWM test at 6 (6M) and 18-month-old (18M).

Between the 21st postnatal day until 6M or 18M, the mice were subjected to one of the three different diet regimens, with no distinction in composition or nutritional value. Animals were fed with continuous pellet hard diet (HD—normal mastication group), or with hard followed by soft diet (HD/SD—reduced mastication group), or with hard diet followed by soft diet, followed by hard diet again (HD/SD/HD—rehabilitated mastication group). Alternated diet regimes were equally distributed along time as follows: 6M mice (3M fed by pellet and 3M by powder diet; 3M HD + 3M SD), 18M mice (9M HD + 9M SD), 6M mice (2M HD + 2M SD + 2M HD), and 18M mice (6M HD + 6M SD + 6M HD). We assumed that powder diet required less masticatory activity as compared to the pellet diet. Each young group (6M) was composed by 10 animals and the aged ones by 12 animals (Table 1).

**TABLE 1:** Number of animals per experimental groups.

Age	Number of animals					
	Impoverished environment (IE)			Enriched environment (EE)		
	HD	HD/SD	HD/SD/HD	HD	HD/SD	HD/SD/HD
6 months (6M)	10	10	10	10	10	10
18 months (18M)	12	12	12	12	12	12

*HD, hard diet/pellet; SD, soft diet/powder food.*

**FIGURE 1:**
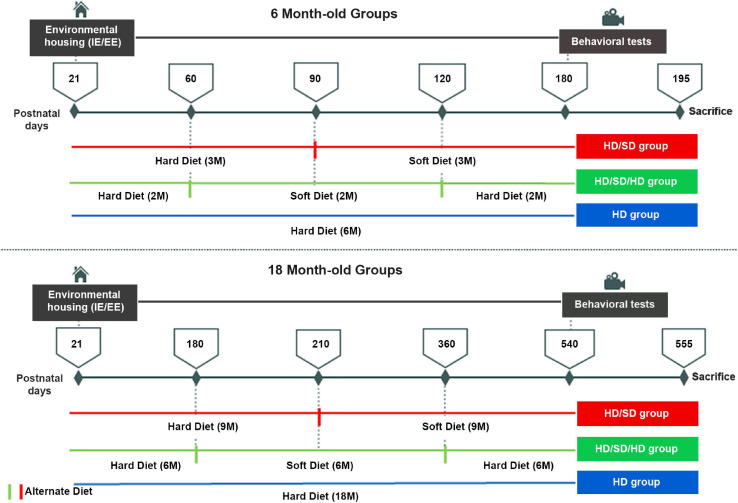
Experimental timeline. Female albino Swiss mice were housed either in impoverished or enriched environments (IE/EE, left) from postnatal day 21 until 6M or 18M, under one of the following diet regimes: hard diet (HD) with continuous pellet diets; sequences of pellet, powder food (soft diet), and pellet food (HD/SD/HD) regimes every 2 for 6M or 6 for 18M, respectively; Alternate pellet and powder diet (HD/SD) regimes for 6 or 18M every 3 or 9M, respectively. All 6M and 18M animals were submitted to the behavioral Morris Water Maze test (right) between 180 and 195 and between 540 and 555 days of life, respectively. IE, impoverished environment; EE, enriched environment; 2M, 2-month-old; 6M, 6-month-old; 9M, 9-month-old; 18M, 18-month-old; HD, hard diet/pellet; SD, soft diet/powder food.

Despite the contrasting masticatory regimes, all animals had free access to diet and to water and were kept in controlled room temperature (23 ± 1°C) with 12 h in a light/dark cycles (clear period: 6:00 A.M.; dark: 6:00 P.M.). From weaning onwards, all animals were kept exclusively in one of the two environmental conditions: impoverished environment of standard laboratory cages [impoverished environment (IE)] or in environmental enriched cages [enriched environment (EE)]. IE was composed of 32-cm × 45-cm × 16.5-cm size polypropylene standard cages for mice, lined with rice straw and covered with metal grids. Each standard cage housed six mice. EE consisted of wire cages with two floors, each of which with 50 cm × 50 cm × 50 cm, equipped with bridges, tunnels, rods, running wheels, and toys. The toys were made of different types of plastic and colors, being displaced or replaced each week, to expose animals to different visual, motor, and somatosensory stimuli. Water and food were offered on the upper and lower floors, respectively. This arrangement forced mice to move from one floor to the other to drink or eat. Each EE cage housed 12 mice (de Siqueira Mendes et al., 2019). Twelve experimental groups were organized: two environments × two ages × three diet regimes. At the end of each time window (6 and 18 M), the animals were submitted to behavioral tests. Figure 1 summarizes the timeline, experimental conditions, and assessed groups.

### Behavioral Tests

Animals were submitted to the MWM test at 6 and 18 M. All the experimental procedures were videorecorded and were performed under the same luminance levels (4–5 cd/m^2^) and time schedules for all groups. The MWM pool (101 cm diameter) was filled with water and maintained at a temperature of 22 ± 2°C. The volume of water was enough to submerge a platform of 22 cm height and 13 cm diameter. Its surface was 1 cm below the water. Platform surface was hidden, with a non-toxic edible black dye dissolved in the water. Cardinal virtual points (North, South, East, and West) were indicated and used to record and plan the experimental session, dividing the pool in quadrants where each mouse was kindly placed to start swimming. The animal started the test in one randomly selected quadrant and platform was placed at the center of one quadrant and remained in the same position until the end of the tests. Three permanent external visual cues were used as reference points to facilitate searching for the submerged platform. Test procedures started after a 60 s single adaptive trial to the apparatus on the first day of testing. If the animal did not find the platform during the available time, it was guided to it and removed from the water once on the platform. After adaptation, all subjects were tested for three consecutive trials per day, on the subsequent 5 days. New entry points were used on each test. Each of the three trials had a maximum duration of 60 s, with a 30 s recovery interval. The task was considered as completed when the animal found and remained on the platform for 10 s. The ANY-Maze Video Tracking System (Stoelting Co^©^.) was used to analyze all behavioral parameters of interest.

### Immunohistochemistry

Immediately after the behavioral tests, all animals were weighed, sacrificed with an overdose of anesthetic, and perfused transcardially with saline followed by 4% paraformaldehyde fixative (in 0.1 M phosphate buffer, pH 7.2–7.4). At the end of the procedure, horizontal 60-μm thick sections were obtained with a Vibratome (Microm^®^ HM 650) and organized as anatomical 1:6 series of sections that were code labeled to assure blind procedures for three-dimensional (3D) reconstruction. One in each six sections of each anatomical series and by each experimental group was immunostained for the selective astrocyte marker, the glial fibrillary acidic protein (GFAP—mouse anti-GFAP monoclonal antibody MAB360, Millipore Int., United States).

### Astrocyte 3D Reconstruction

3D reconstructions of astrocytes were performed using a Neurolucida computer software (MBF Bioscience Inc., Frederick, MD, United States), running in a computer connected to a NIKON Eclipse 80i (Nikon, Japan) microscope, equipped with a motorized plate (MAC6000, Ludl Electronic Products, Hawthorne, NY, United States). The astrocytes were reconstructed with an oil immersion objective, plan fluore, high magnification (100×), high resolution, and small depth of focus (Nikon, NA 1.3, DF = 0.16 μm). To define the limits of the area of interest, ensuring that selected cells were inside the outer third of the molecular layer of the dentate gyrus, a high resolution 4× objective was used (Nikon, NA 0.13, DF = 17.2 μm). After establishing a contour outline draw, the molecular layer of the dentate gyrus (our area of interest) was divided into three equal parts to delineate the anatomical limits of its outermost one-third. Thirty cells per animal were reconstructed digitally. As we had more than one section having the area of interest to assess the number of cells to be reconstructed in each section, we estimated the total area of interest and used the relative proportion of each section to estimate the number of astrocytes to be reconstructed per section. To guarantee that all regions of the area of interest had the same probability to contribute to the sample, a grid of 50 μm × 50 μm was placed over the area of interest. One single astrocyte within a squared box of 50 μm × 50 μm was selected for 3D reconstruction. Thus, the selected astrocytes were symmetrically distributed over the external third of the dentate gyrus molecular layer. The one that was located more centrally and that unequivocally belonged to the outer one-third molecular layer of the gyrus was chosen. Vascular astrocytes were excluded. Incomplete immunolabeling or astrocytes with cut branches were discarded from the analysis.

Wherever astrocytes were absent in the chosen box, we selected the one nearest to its borders for reconstruction. As our sample consisted of 60 animals and 30 cells per animal were reconstructed, a total number of 1,800 astrocytes were analyzed.

### Statistical Analysis

#### Behavior Analysis

Each animal realized three daily trials over five testing days, and a daily mean value of escape latency was estimated. Likewise, mean traveled distances were calculated (total traveled distance and distance traveled in the opposite quadrant to the hidden platform). An average of swimming speed was also calculated.

From daily averages of escape latencies, an index of learning and memory was established. This index was calculated by the ratio between escape latencies of the first day test (neglecting adaptation phase) and escape latencies of subsequent days (second to fifth days) of test based on the following equation: CI = (*L*_1_ − *L*_*N*_)/(*L*_1_ + *L*_*N*_), where CI was the contrast index expressing the learning rate, *L*_1_ the mean of escape latency to find the platform on the first day test, and *L*_*N*_ the mean of escape latency to find the platform on subsequent days (second, third, fourth, or fifth days). From this analysis, four contrast values were obtained for each animal (*L*_1_ and *L*_2_, *L*_1_ and *L*_3_, *L*_1_ and *L*_4_, and *L*_1_ and *L*_5_ ratios). The contrast index was already applied in previous studies using the MWM to normalize the learning curve (Torres et al., 2006; Diniz et al., 2010). Such equation was then systematically applied to all training days using the mean daily latency value based on the three trials. When CI > 0 was obtained, it meant that the animal found the platform in a shorter time when compared to the first day of learning test. Values of CI < 0 revealed longer time spent to find platform, and CI = 0 meant that no differences between latency escapes were found.

From this analysis, it was possible to determine which animals showed consistent learning during trials. Consistent learning was defined when the animal had CI > 0 in at least three comparisons from the four assessed (*L*_1_ and *L*_2_, *L*_1_ and *L*_3_, *L*_1_ and *L*_4_, and *L*_1_ and *L*_5_), or in two comparisons in the fourth and fifth training days (*L*_1_ and *L*_4_, *L*_1_ and *L*_5_). If an animal did not meet one of these criteria, it was excluded from analysis because the learning criterion was not reached. Aligned to this, we expressed the CI in percentage values, as the learning rate. The highest contrast value among animals was selected from all experimental groups regardless of diet, age, or environment as 100%. Thus, all other percentage values were estimated as relative proportions of the best performance, as follows: C (%) = (CI × 100)/CI max, where C (%) was the contrast index of learning rate expressed in percentage values, CI the contrast index that expressed individual learning rate, and CI max the one corresponding to the highest value of CI, representing the best performance obtained among sample individuals.

To compare the learning rates of the experimental groups, we used the fourth day of training results. The reason for this was that all groups revealed on the fourth training day, the largest number of animals with a learning index ≥60%. For this comparison, the five best performances of each experimental group were adopted. Thereafter, three-way ANOVA test was applied, followed by the Tukey posttest (honestly significant difference) to analyze the learning rate in percentage values, total distance traveled, distance traveled in the opposite quadrant, and the average swimming speed. Significant differences between groups were set at 95% confidence level.

#### Three-Dimensional Reconstruction

All astrocytes were analyzed for several morphometric features of the cell soma and of the tree branches using 3D reconstruction analysis with Neurolucida Explorer computer program (MBF Bioscience Inc., Frederick, MD, United States). *Z*-axis retraction was corrected for shrinkage of 75% due to the histological processing, as previously recommended (Carlo and Stevens, 2011). In the *X*- and *Y*-axes, the retraction was minimal, and there was no need for correction. Each astrocyte was analyzed several times using the following morphometrical features: area, perimeter, Feret max and min diameters, aspect ratio, compactness, convexity, shape factor, sphericity and solidity related to the cell soma, and number of segments, segments per millimeter, number of trees, total branch length (μm), average branch length (μm), tortuosity, average branch surface area (μm^2^), total branch volume (μm^3^), primary branch base diameter (μm), planar angle, fractal dimension, convex hull [perimeter (μm), area (μm^2^)], 2D, surface area [(μm^2^), 3D, and volume (μm^3^)], vertex analysis, and morphological complexity, relatively to the cell branches. Only morphometric features with multimodal distribution (with at least two modes) were used to classify cells using hierarchical cluster analysis (Schweitzer and Renehan, 1997). For this, we estimate the multimodality index (MMI). The MMI was estimated based on asymmetry and kurtosis of our sample (150 cells from each experimental group), according to the following equation: MMI = (M3^2^ + 1)/[M4 + 3 (*n* − 1)^2^/(*n* − 2) (*n* − 3)], where M3 was the skewness, M4 the kurtosis, and n the sample size (Kolb et al., 1994; Schweitzer and Renehan, 1997). Morphometric features showing MMI > 0.55 were used for classification (Schweitzer and Renehan, 1997).

Then, the same data were submitted to discriminant analysis, a multivariate statistical procedure was used to discriminate and classify objects. Discriminant analysis allowed to identify which of morphometric variables most contributed to the cluster’s formation. All analyzes were performed using the Statistica 7.1 software (Copyright^®^ StatSoft, Inc., 2005) and Bioestat 5.3.

## Results

### Morris Water Maze Performance

**FIGURE 2:**
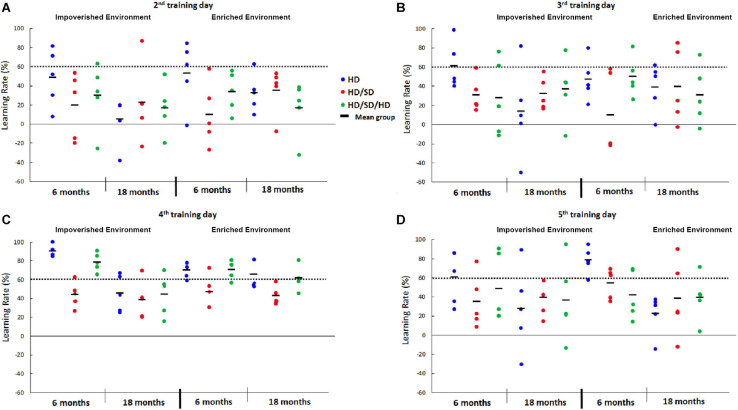
Learning rate percentage (%) from the 2nd to 5th day in the Morris Water Maze test under different diet regimes (HD, HD/SD, and HD/SD/HD), environmental conditions (impoverished and enriched) and age (6 and 18 months). Graphic representation of individual performances (colored solid circles) and group means (dark lines between solid circles). **(A)** Graphic representation of individual learning rates at the 2nd testing day. **(B)** Graphic representation of individual learning rates at the 3rd testing day. **(C)** Graphic representation of individual learning rates at the 4th testing day. **(D)** Graphic representation of individual learning rates at the 5th testing day. HD, hard diet/pellet food and SD, soft diet/powder food.

**FIGURE 3:**
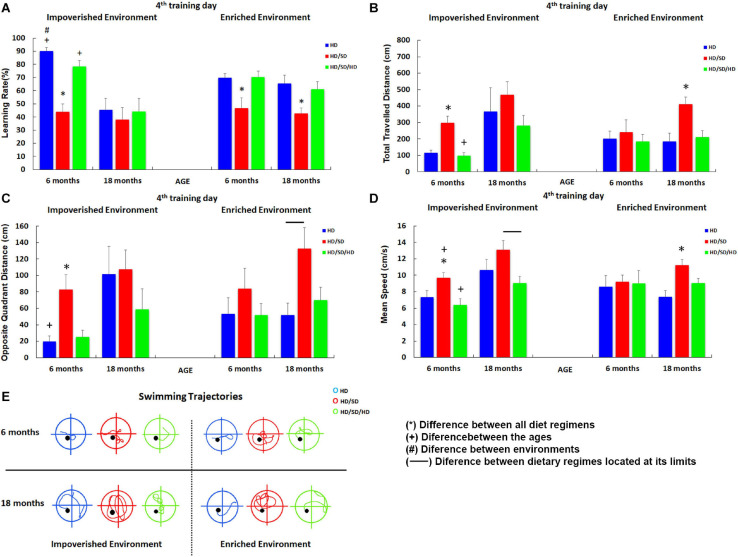
Influence of environment (impoverished and enriched), age (6 and 18-month-old) and diet regimens (HD, HD/SD, and HD/SD/HD) in the learning rates by the Morris Water Maze protocol at the 4th testing day. **(A)** Learning rates in percentage (%) for each experimental group. **(B)** Total distance (cm) traveled by each experimental group. **(C)** Distance (cm) traveled in the opposite quadrant of the platform. **(D)** Swimming speed (cm/s). **(E)** Swimming trajectories representative of each group. Trajectories correspond to the average distance closer to that of the group. Results are expressed as mean ± standard error. (*) Significant differences between all diet regimes; connector lines indicate significant differences between dietary regimes; (+) Significant differences between ages; (#) significant differences between environments. HD, hard diet/pellet food and SD, soft diet/powder food.

We obtained the learning rates of each animal over the 5 days of behavioral tests, and the fourth day of training was the one with the highest number of animals with a learning rate ≥60% for all experimental groups. Comparative rate learning represented as percentage (%) of the best performance was estimated for each animal of each experimental group over the testing days, and the respective means are depicted in Figure 2. Influence of the three diet regimens, control, reduced, and rehabilitated (HD, HD/SD, and HD/SD/HD) at different ages (6M and 18M) and environments (IE and EE), on the fourth day of testing, are shown in Figure 3.

As compared with HD, 6M HD/SD mice with IE and EE showed statistically significant lower values of learning rate (Figure 3A). In contrast, the rehabilitated HD/SD/HD young group recovered learning rates up to control HD levels. Aged mice subjected to long-term environmental impoverishment independent of diet regime showed statistically significant reduction in spatial learning and memory, whereas long-term environmental enrichment in combination with rehabilitation of masticatory activity (HD/SD/HD) recovered spatial learning rate up to control HD levels. Therefore, from this data set emerges the concept that masticatory activity reduction decreases learning rate and spatial memory while masticatory rehabilitation in combination with environmental enrichment seemed to recover all losses imposed by the reduction in mastication. It is also worth to note that aging and IE, when acting together, significantly decrease the learning rate. The influence of masticatory activity [*F*_(__2_._48__)_ = 17.0, *p* < 0.000003], age [*F*_(__1_._48__)_ = 20.9, *p* < 0.000034], and the interaction between age and environment [*F*_(__1_,_48__)_ = 8.98; *p* < 0.0043] on the learning rate were evident by the three-way ANOVA tests. Detailed information for each experimental group including Tukey posttest results is available in Supplementary Material 1 Tables 1, 2.

As the reduction in MWM escape latency may simply be a consequence of increased swim speed over the period and/or significant changes on thigmotaxic strategy from the first days of training, we further analyzed other parameters. Relative to the total distance traveled in centimeters (Figure 3B), paired comparisons showed that the HD/SD IE 6M group (deprived group maintained at IE and 6M) covered longer distances compared to the HD 6M and HD/SD/HD 6M groups in IE, while the young HD/SD/HD rehabilitated group presented decreased levels than the aged one. This same effect was observed when comparing HD/SD 18M with HD 18M and HD/SD/HD 18M, all from EE. The three-way ANOVA for these data showed that the total distance traveled was influenced by age [*F*_(__1_,_48__)_ = 12.4, *p* < 0.00096] and by masticatory changes [*F*_(__2_,_48__)_ = 7.24, *p* < 0.0018]. As expected, the best performances in learning rates were associated with the shorter distances.

Significant influences of age [*F*_(__1_._48__)_ = 8.29, *p* < 0.0059] and masticatory regime [*F*_(__2_._48__)_ = 7.24; *p* < 0.0018] were found in distance traveled in the opposite quadrant to the platform (Figure 3C). At 6M, both HD and HD/SD/HD control and rehabilitated groups showed, on average, the lower values of distance traveled in opposite quadrant, whereas HD/SD showed the greater one. Moreover, HD/SD old mice showed greater values as compared to HD and HD/SD/HD 18M groups.

In the paired comparisons (Figure 3D), when we considered the IE condition, the HD/SD 6M group swam faster than the HD 6M and HD/SD/HD 6M groups. However, this group was slower than the HD/SD 18M group. In turn, the HD/SD/HD 18M group swam faster than the HD/SD/HD 6M one, but the former ended up with a slower swimming than the HD/SD 18M group. Despite the significant differences found in IE groups, swimming speed did not change in EE animals, except for HD/SD 18M, which swam faster than HD 18M and HD/SD/HD 18M. It is important to point out that swimming speed was affected by age [*F*_(__1_,_48__)_ = 8.94, *p* < 0.004] and by masticatory change [*F*_(__2_._48__)_ = 7.86, *p* < 0.0011] with a significant interaction between environment and age conditions [*F*_(__1_._48__)_ = 6.38, *p* < 0.015]. Interestingly, in a paired analysis (Figures 3C,E), we found that control and rehabilitated young groups maintained is an impoverished environment (HD IE 6M and the HD/SD/HD IE 6M groups) swim less than the HD/SD IE 6M in the opposite quadrant, which suggests a better strategy to find the platform for the first two groups in relation to the one with masticatory reduction. The same pattern occurred for the HD IE 6M vs. HD IE 18M (control groups at different ages) and HD EE 18M vs. HD/SD EE 18M (masticatory activity deprivation at aging animals).

All mean values and standard error values related to total distance traveled, distance in the opposite quadrant, and mean values of swim speed, as well as significant differences for paired samples, obtained after the Tukey test (DHS) and by each experimental group, are available in Supplementary Material 1 Tables 3–8.

Taking these findings together, the learning rates seem to be inversely related to the distance traveled. Therefore, the experimental groups showing the best performances in the MWM test also revealed the shorter swimming trajectories. Because their swimming speed was also lower, we suggest that the effects of reduced masticatory activity, environmental impoverishment, and aging on the learning rate of female mice in the MWM task are due to cognitive changes and cannot be explained by the differential somatomotor effects. The results also suggest that the rehabilitation of masticatory activity in animals previously submitted to the reduction in this activity can benefit both the young and elderly mice in the spatial learning and memory test performance.

### Body Weight and Diet Regimens

To investigate the influence of diet regimes on body weight, all mice were weighed shortly after completing the behavioral tests. The one-way ANOVA test (Student’s *t* posttest, *p* < 0.05) revealed a statistically significant difference between the weights of the IE animals in which the HD/SD 6M weighed significantly less than HD 6M and HD/SD/HD 6M groups. Furthermore, at IE condition, HD 18M group weighed significantly more than HD/SD 18M and HD/SD/HD 18M ones. Based on these differences in body weight of some experimental groups, we additionally tested their potential correlation with learning rate using Pearson’s linear correlation. Only a significant correlation was obtained between body weight and animal learning in the MWM test for the group HD/SD in IE (Supplementary Material 1 Table 11). However, since no other simple linear correlations were detected, we believe that body weight was not responsible for the significant differences we found. Details of mean values and standard error for body weight for each experimental group and statistical testing for the paired samples and values for Pearson correlation analysis significance are depicted in Supplementary Material 1 Tables 9–11.

### Morphometric Analysis of Astrocytes

Estimation of the multimodal index (MMI) for each of the 30 astrocyte morphological variables showed that morphological complexity with MMI > 0.55 was found in all groups, except in the HD IE 18M group. In addition, complexity was also indicated by discriminant analysis as the morphological variable that most contributed to cluster formation (Supplementary Material 2 Table 1 shows details for other morphological variables with MMI > 0.55 and discriminant analysis results).

**FIGURE 4:**
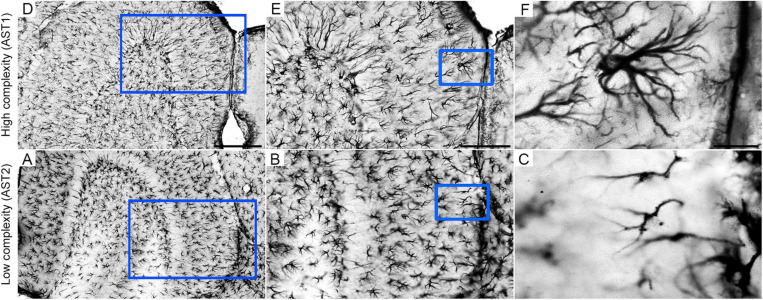
Low **(A,D)**, middle **(B,E)**, and high **(C,F)** power photomicrographs of GFAP-immunolabeled astrocytes to illustrate high (AST1) and low (AST2) morphological complexities in the external one third of molecular layer of mouse dentate gyrus. Blue squares identify the anatomical region from where pictures of illustrated cells were taken. Scale bars: A/D = 250 urn; B/E = 125 urn; C/F = 25 urn. Astrocyte AST1 (high morphological complexity); AST2: Astrocyte AST2 (low morphological complexity).

As HD IE 18M (aged control group at impoverished conditions) was the only one that did not show MMI > 0.55 for morphological complexity, the hierarchical cluster analysis used to classify astrocytes of this group was based on the cell soma morphometric features, which included convexity and solidity as the multimodal morphological variables detected in this group. After hierarchical cluster and discriminant analysis, we measured potential influences of diet regime, age, and environment on morphological complexity of astrocytes of all experimental groups. The cluster analysis of each experimental groups could classify astrocytes into two main astrocytes morphological groups, named AST1 and AST2, according to their greater and lower complexity, respectively (Diniz et al., 2016). Of course, AST1 and AST2 are only the extremes of a kaleidoscope of morphological changes that we found in our sample of 1,800 astrocytes. Thus, to illustrate the concept of morphological complexity differences, photomicrographs of astrocytes from the molecular layer of the dentate gyrus are included in Figure 4, where examples of higher and lower morphological complexities are shown.

**FIGURE 5:**
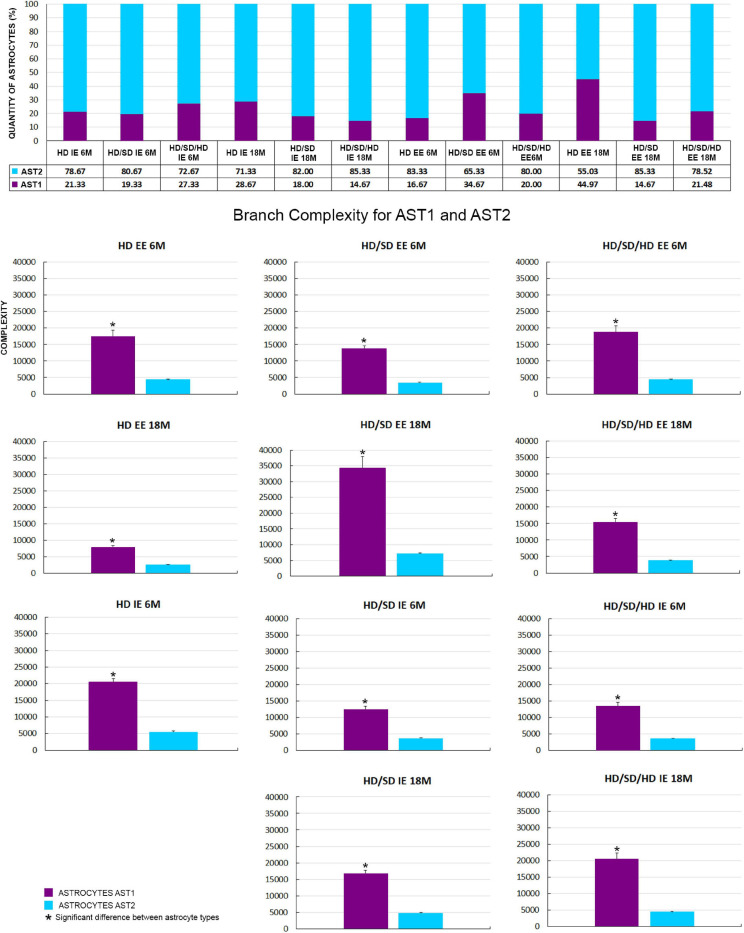
**(Top)** Graphical representation of astrocyte number (absolute values and percentage) by cellular phenotype (AST1 and AST2) for each diet regime (HD, HD/SD, and HD/SD/HD), environment (impoverished – IE and enriched – EE) and age of 6 (6M) and 18 months (18M), on the top. **(Bottom)** Comparison of mean branch complexity values and corresponding standard errors to illustrate differences between AST1 and AST2 for all experimental groups. HD, hard diet/pellet food and SD, soft diet/powder food. (*) Indicates *p* < 0.05. IE, impoverished environment; EE, enriched environment; 6M, 6-month-old; 18M, 18-month-old; 9M, 9-month-old; HD, hard diet/pellet; SD, soft diet/powder food; AST1, astrocyte with high morphological complexity; AST2, astrocyte with low morphological complexity.

Although all the experimental groups revealed the presence of these two AST1 and AST2 morphological phenotypes of astrocytes, their proportion was not homogeneous across groups. Numbers of AST2 were always prevalent relative to those of AST1 cells, with an exception for HD EE 18M, where similar distribution was observed (Figure 5).

Thus, given the fact that experimental groups showed two AST1 and AST2 morphological subtypes of astrocytes, it became essential to investigate whether there were significant differences between their morphological complexities inside the same experimental groups. For that, the samples were compared two by two using the Student’s *t*-test, adopting a 95% confidence level (Figure 5). Independently of diet, age, or environment, the complexity of AST1 morphotype was significantly higher than that of AST2 in all cases, except in the HD IE 18M group (not shown at Figure 5). The mean and standard error values for the morphological complexity of each phenotype per experimental group, and the paired samples significances, are available in Supplementary Material 2 Table 2.

**FIGURE 6:**
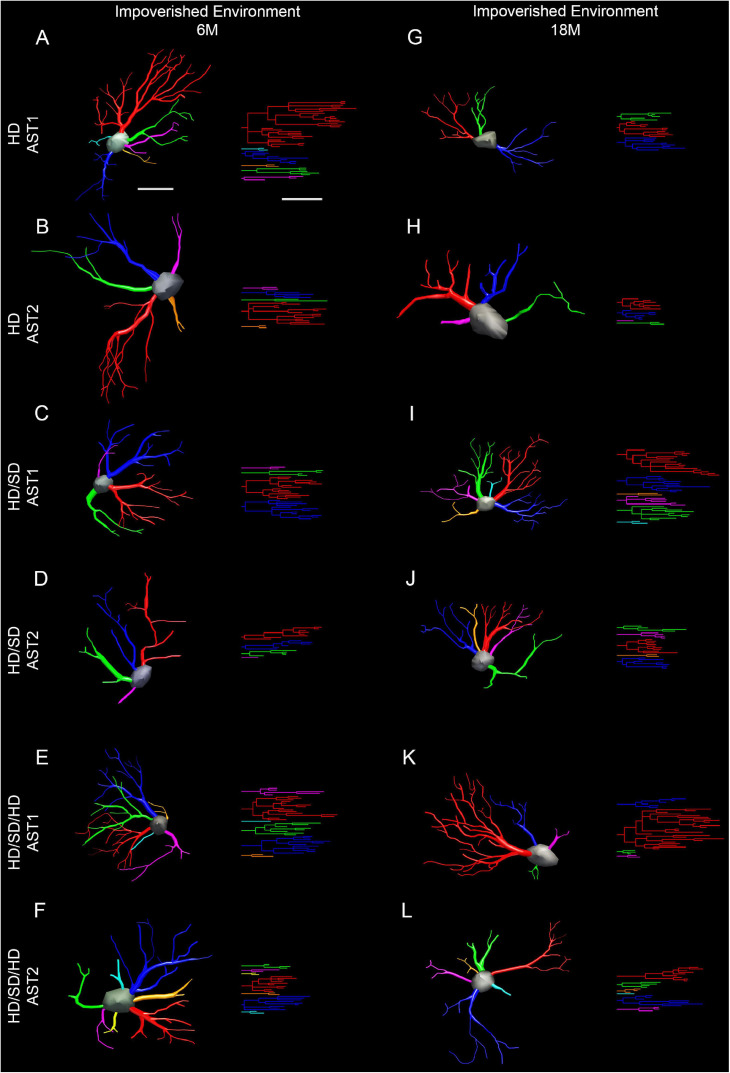
Three-dimensional reconstruction of the morphological phenotypes of astrocytes located in the outer 1/3 of the molecular layer of dentate gyrus, in *Mus musculus* animals, and respective dendrograms of each astrocytic phenotype per experimental group maintained at impoverished environment, reconstructed and analyzed through the Neuroexplorer software (MicroBrightField). To choose the representative cell of each group, the distance matrix was used to obtain the sum of the distances of each cell in relation to all the others. We considered the cell better representing each group as having at the least the sum of distances. Branches originating from the same parental trunk (primary branch) are shown with the same color. **(A–F)** 6M; **(G–L)** 18M. Cell bar = 15 μm; dendrogram bar = 15 μm. 6M, 6-month-old; 18M, 18-month-old; HD, hard diet/pellet; SD, soft diet/powder food; AST1, astrocyte with high morphological complexity; AST2, astrocyte with low morphological complexity.

**FIGURE 7:**
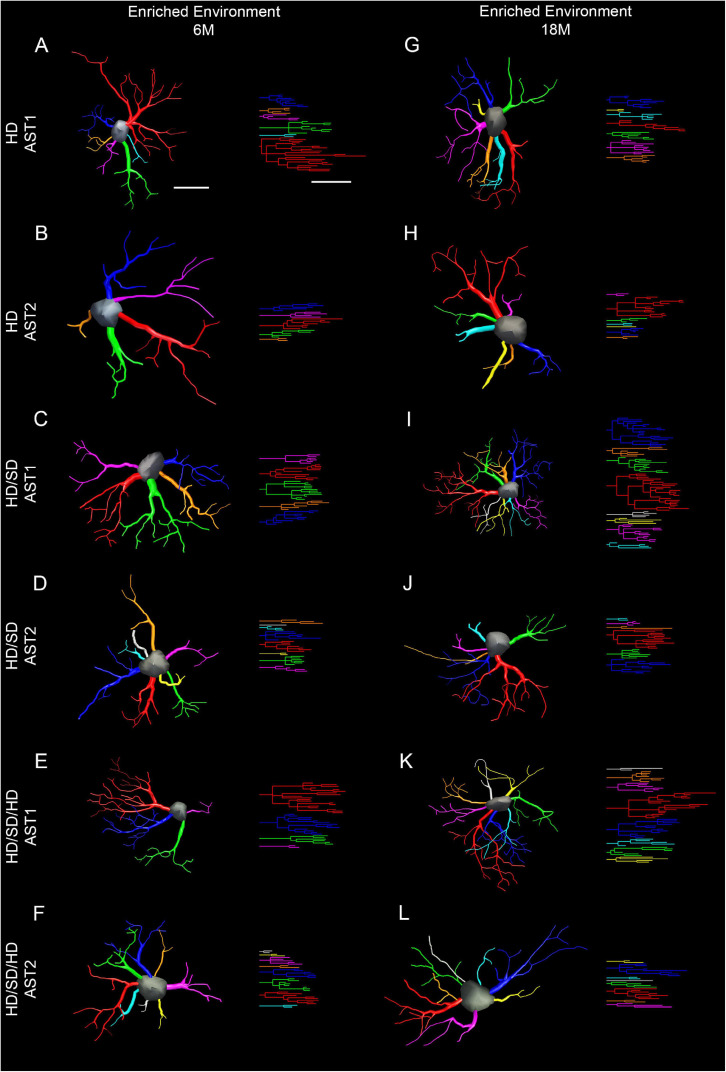
Three-dimensional reconstruction of the morphological phenotypes of astrocytes located in the outer 1/3 of the molecular layer of dentate gyrus, in *Mus musculus* animals, and respective dendrograms of each astrocytic phenotype per experimental group maintained at enriched environment, reconstructed and analyzed through the Neuroexplorer software (MicroBrightField). To choose the representative cell of each group, the distance matrix was used to obtain the sum of the distances of each cell in relation to all the others. We considered the cell better representing each group as having at the least the sum of distances. Branches originating from the same parental trunk (primary branch) are shown with the same color. **(A–F)** 6M; **(G–L)** 18M. Cell bar = 15 μm; dendrogram bar = 15 μm. 6M, 6-month-old; 18M, 18-month-old; HD, hard diet/pellet; SD, soft diet/powder food; AST1, astrocyte with high morphological complexity; AST2, astrocyte with low morphological complexity.

To choose a representative cell from each group, we used the distance matrix to obtain the sum of the distances of each cell in relation to all others. We assumed that the cell that better represents each group would have the least sum of distances. Thus, the matrices were constructed with the combination of all cells of a given group, followed by the weighted calculation of a scalar Euclidean distance between the cells using morphometric variables in which MMI was higher than 0.55. In this way, Figures 6 and 7 show 3D reconstructions and corresponding dendrograms for each morphotype of representative astrocyte per experimental group.

**FIGURE 8:**
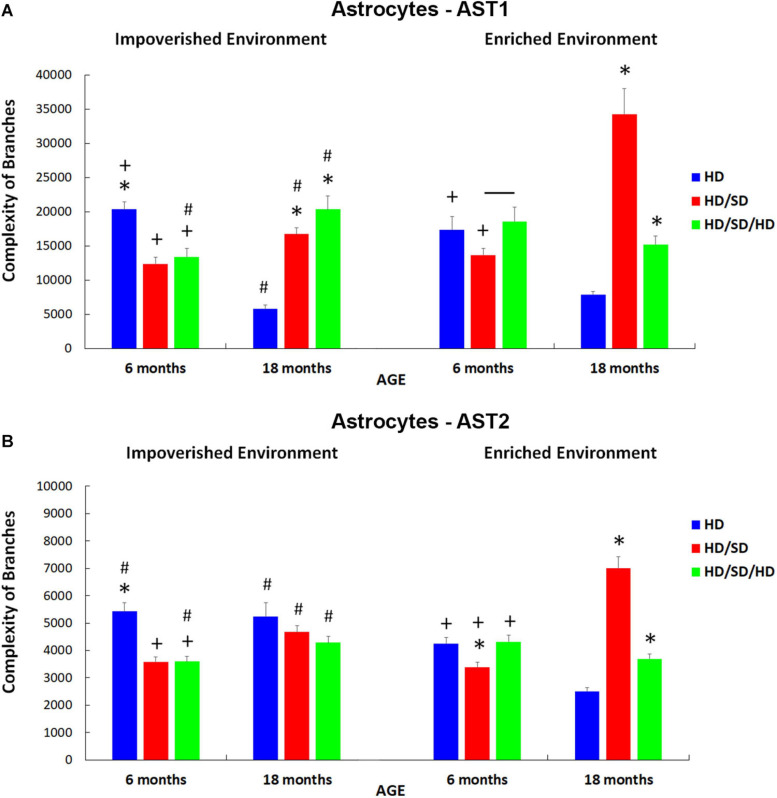
Branch complexities of AST1 **(A)** and AST2 **(B)** for each diet regime (HD, HD/SD, and HD/SD/HD), and environment (impoverished and enriched), at 6 and 18 months of age. Results are expressed as mean + standard error. (*) Significant differences between all diet regimens; Connector lines indicate significant differences between dietary regimes; (+) Significant differences between ages; (#) Significant differences between environments. HD, hard diet/pellet food; SD, soft diet/powder food; AST1, astrocyte with high morphological complexity; AST2, astrocyte with low morphological complexity.

Following the analysis that allowed the identification of significant differences between AST1 and AST2, we assessed differences between groups to identify potential alterations of AST1 and AST2 subtypes by doing analysis of variance and Student’s *t*-test (Figure 8). Statistical details and significant differences between AST1 and AST2 are indicated in Supplementary Material 2 Tables 3, 4.

For AST1 subtype (Figure 8A), it was noted that in the control group, regardless of the environment, astrocyte cell complexity was reduced with aging (HD IE 6M vs. HD IE 18M and HD EE 6M vs. HD EE 18M). Note that in the context of environmental stimulation, these differences imposed by age had less impact when compared with the differences between HD IE 18M vs. HD EE 18M and HD IE 6M vs. HD EE 6M (comparison between control groups at different environments) (Figure 8A). In this sense, enriched environment seems to reduce the effects of aging on the morphological complexity of AST1 astrocytes.

In addition to the findings already obtained, it was essential to examine the pattern of such differences between the groups that suffered changes in masticatory activity and the control group. In the case of 6M IE (Figure 8A), deprivation in chewing led to a reduction in the complexity of astrocytes (HD vs. HD/SD (Figure 8A).

On the other hand, unlike that observed in 6M animals, aged mice that suffered rehabilitation in masticatory activity (HD/SD/HD) showed a great increase in the complexity of branches of AST1, comparing IE vs. EE. Interestingly, in the EE, the same differences were not observed, and the only difference obtained was between HD/SD EE 6M vs. HD/SD/HD EE 6M animals (Figure 8A).

It is important to highlight that environmental enrichment in combination with masticatory rehabilitation, although not reaching the morphological complexity of control levels (HD 18M vs. HD/SD/HD 18M), induced significant morphological changes as compared with aged mice under masticatory reduction (HD/SD 18M vs. HD/SD/HD 18M). Indeed, the combination EE and rehabilitation showed an inversion of the observed pattern in comparison the HD/SD IE 18M vs. HD/SD/HD IE 18M.

Here, it is important to point out that a correlation was not found between the morphological complexity of AST1 and learning rates (Figure 3), which supports a view that the morphological changes are related to distinct adaptive plasticity responses within these morphotypes.

When analyzing the effects of the experimental conditions on the complexity of AST2 (Figure 8B), the similarity of the findings described for AST1 was readily recognized. However, the group of elderly mice kept in the standard environment did not follow the same pattern because, unlike what happened with AST1, AST2 cells did not show a reduction in the complexity of their branches compared to HD 6M animals kept in the same conditions (IE). Instead, the complexity remained unchanged in old IE animals (HD vs. HD/SD and HD vs. HD/SD/HD), and this was also observed among the groups with masticatory changes (HD/SD vs. HD/SD/HD).

Another particularity in the analysis of AST2 when considering the EE condition (Figure 8B) was the difference found between HD/SD 6M, HD 6M, and HD/SD/HD 6M groups, with the group with less masticatory activity (HD/SD) showing a lower branch complexity. In addition, the HD/SD/HD 6M with EE revealed greater complexity than the HD/SD/HD 18M with EE, as well as the HD 6M with IE condition, when compared to HD 6M with EE one.

## Discussion

In the present work, we tested whether the reduction in masticatory activity and the sedentary lifestyle aggravated cognitive decline, and if masticatory rehabilitation and an active lifestyle led to a recovery or delay of cognitive losses trying to track an association with astrocyte morphological changes in the molecular layer of dentate gyrus. We found that reduced mastication acting together with IE impaired spatial learning and memory, while masticatory rehabilitation and EE mimicking an active lifestyle and acting together recovered all mice cognitive losses. Although non-linear correlations were found between learning rate and astrocyte morphological changes, the long-term EE applied to the masticatory-deprived group reversed the shrinkage effect associated with the reduced mastication and IE, with significant differential increase in the morphological complexities of AST1 and AST2.

Consistent with our findings, a recent study demonstrated that mice fed with solid diet significantly reduced escape latency in MWM test, suggesting that consumption of a solid diet is related to improved performances when spatial memory is requested (Akazawa et al., 2013). Further studies using other behavioral assays, including MWM (Onozuka et al., 1999, 2000, 2002; Watanabe et al., 2001b, 2002; Kubo et al., 2005; Tsutsui et al., 2007; Frota de Almeida et al., 2012), passive avoidance (Kushida et al., 2008), and radial labyrinth (Kato et al., 1997; Yamamoto and Hirayama, 2001; Yamazaki et al., 2008) reported that the provision of a long-term powdered diet or molar tooth extraction resulted in learning and memory deficits (Kubo et al., 2010a).

The murine fast-aging model SAMP8 reaches adult maturity at 6 M, with memory and learning deficits apparent in a variety of behavioral tests (Yagi et al., 1988; Ohta et al., 1989; Flood and Morley, 1993; Miyamoto et al., 2005). In these mice, restriction of masticatory activity was shown to accelerate age-related decline changes (Kubo et al., 2010a). This subject was recently reviewed by Ono et al. (2010) and Kubo et al. (2010b). Given these data and our current findings, it will be important for investigators to explore the influence of diet on cognition, considering the consistency of food stuffs that may be employed. Many high-fat diets are much softer that the control diets used in the present study, and as a consequence, mastication may be a hitherto unrecognized confound in outcomes both in the past and future.

Our findings are in line with a previous report (Frota de Almeida et al., 2012) and demonstrate that the rehabilitation of masticatory activity of mice subjected to a reduction in mastication may recover spatial learning losses at adulthood (6M). However, we found that aged mice (18M) required a combination of EE and masticatory rehabilitation to achieve similar effects. As hypothesized, the ability to learn and remember the location of the hidden platform became worse as the animals aged and was aggravated by the IE mimicking a sedentary lifestyle.

Because MWM is a hippocampal-dependent task, and functional integrity of the hippocampal structure is critical for spatial learning and memory, we hypothesized that rehabilitation of masticatory activity in young adult mice, and a combination of EE and masticatory rehabilitation in aged ones, might rescue the hippocampal homeostatic conditions, thus restoring the mnemonic function of spatial location performed by the hippocampus. In agreement, aged mice maintained in enriched cage from weaning exhibited relatively intact spatial learning in the MWM task, suggesting that consolidation and recovery mechanisms for these memories were spared under enriched conditions (Diniz et al., 2010; Obiang et al., 2011).

The component of physical activity, such as voluntary exercise in the running wheel in enriched cages, has been associated with improved cognitive function through effects on neural processes, such as hippocampal neurogenesis (van Praag, 2008), cerebral angiogenesis (Kerr et al., 2010), increased expression of neurotrophic factors (Lista and Sorrentino, 2010), gene expression (Kohman et al., 2011), and neuritic growth (Wu et al., 2008). More recently, the influence of institutionalization on elderly, sedentary human and living in IE at long-term care institutions was investigated. The results from neuropsychological assessment, as compared with the control group of elderly living in a community, i.e., enriched conditions, showed significantly higher scores in language and cognitive tests among participants living in the community. Coherent, institutionalized elderly patients subjected to the 6M intervention program of cognitive and multisensory stimulation, corresponding to EE, significantly improved their performance on neuropsychological tests (De Oliveira et al., 2014).

Regarding the morphological changes observed in astrocytes, it is well known that they are highly heterogeneous in shape and function and demonstrate a remarkable adaptive plasticity to guarantee functional maintenance of the CNS across the life span (Augusto-Oliveira et al., 2020; Pestana et al., 2020). Astrocytes are strongly integrated into neural networks and act within the context of the neural tissue controlling CNS homeostasis at all levels of organization (Verkhratsky and Nedergaard, 2018).

Related to the morphological results of the astrocytes located in the outer third of the molecular layer of the dentate gyrus, taken together with the studied variables (environment, age, and masticatory activity), we recognize that regardless of their interactions or effects, two morphological phenotypes remained apparent in all experimental groups. Because AST1 and AST2 were differentially affected by age, environment, and mastication, exhibiting contrasting responses, it is reasonable to assume that the changes in their morphotypes may have distinct functions in each situation. It is important to highlight that no simple correlation was found between the morphological complexity of astrocytes and diet, environment, or age, indicating differential effects of these variables on AST1 and AST2 morphological complexities and suggesting distinct adaptive plasticity of these morphotypes. Regarding the effects on the learning rate and spatial memory, it became apparent that during housing under IE, the ability of learning was reduced by SD (HD/SD) in young but not old mice, whereas it was reversed by HD (HD/SD/HD). During housing under EE, the ability of learning was reduced by SD (HD/SD) in both young and old mice, whereas it was reversed by HD (HD/SD/HD).

Relatively to morphology, the important and unique adaptive astroglial ability seems to have its origin in the development period. Fundamentally different from the neurons, which at birth are usually postmitotic (indivisible), astrocytes maintain their proliferative capacity. In addition, epigenetic regulation of astroglia seems to be equally different, as indicated by the glial methyloma (set of modifications from DNA methylation processes), where they remain more immature and unstable during adult life (Lister et al., 2013). Because of such astrocytic plasticity, we suggest that the combination of mastication, environment, and age acting together can induce adaptive functional responses associated with the structural changes as those described in the present work, showing different manifestations from the perspective of age, environmental conditions, and masticatory activity. In particular, there is evidence that astrocytes are particularly sensitive to aging (Landfield et al., 1977; Lindsey et al., 1979; Rodríguez et al., 2014), masticatory imbalances (Kato et al., 1997), and environmental changes (Viola et al., 2009; Diniz et al., 2016), without reference to the combination of the three factors. The notion that aging and masticatory changes may cause similar cellular and molecular alterations, is suggested by other studies describing astrocytic hypertrophy in the hippocampal area CA1 (Onozuka et al., 2000; Watanabe et al., 2001a), probably as a consequence of astrocyte activation by proinflammatory cytokines (Ono et al., 2010), with no reference regarding its morphometry and/or pattern and complexity.

It has been pointed out that the number of reactive astrocytes increases with normal aging and that the perturbed function of specific astrocyte subpopulations may lead to disease (Clarke et al., 2018; Pestana et al., 2020). In addition, the suppression of astrocyte function, or the gain of an inflammatory profile by cellular senescence, was proposed to be implicated in age-associated neurodegenerative disorders (Cohen and Torres, 2019).

In the aging hippocampus, even in the absence of masticatory imbalance or neurological diseases, astrocytes present a more reactive phenotype (Godbout and Johnson, 2009; Boisvert et al., 2018). We may speculate how neuroinflammation might contribute to the complexity of the response at different ages in the groups with altered masticatory activity. Such speculation would be in keeping with studies that used murine models of rapid aging. In SAMP8 models, young, adult, and old mice, after bilateral molar extraction, demonstrated that circulating levels of corticosterone increased with age, and these levels were significantly higher in adult and old mice that had their molars extracted, when compared with age-matched controls (Iinuma et al., 2014). Thus, this chronic stress in elderly SAMP8 mice with tooth loss could act as a chronic stressor, capable of causing a significant increase in circulating corticosterone levels (Furuzawa et al., 2014). In the present study, we found that aging, independently of environment, contributed to a reduction in the branching complexity (HD 6M IE vs. HD 18M IE and HD EE 6M vs. HD EE 18M). In contrast, AST2 from animals raised in impoverished conditions (HD IE 6M vs. HD IE 18M) did not evidence complexity reduction, thus reinforcing that the concept astrocytes may have multifaceted plastic reactions depending on the CNS region and the interventions performed.

In the IE young animals with masticatory changes, astrocytes were morphologically less complex than in the control group (HD 6M vs. HD/SD 6M and HD 6M vs. HD/SD/HD 6M), independently of the phenotype. Previous studies based on GFAP expression demonstrated that the extraction of the molar teeth accentuated astrocyte hypertrophy and cell density and that such effects were bigger when the condition persisted (Onozuka et al., 2000; Watanabe et al., 2001a). In this context, although astrocytes exhibit morphological heterogeneity comparable to that of neurons, the detailed mapping of their gene expression and functional differences in different parts of the brain is far from trivial, since no universal astroglial cell marker has yet been discovered (Verkhratsky and Nedergaard, 2018).

When astrocytes are involved in neurovascular control (Kuga et al., 2011) and contribute to blood flow regulation, a reduction in GFAP immunolabeling is documented in older animals (Verkhratsky and Nedergaard, 2018). It is reasonable to assume that the hippocampal blood flow may be altered in 18M animals, justifying the decreased complexity of astrocytic branches in our aged control groups. This decrease in GFAP immunolabeling was suggested to be secondary to a reduced neural activity by aging in the mouse temporoammonic projection pathway (Bartesaghi and Gessi, 2003; van Groen et al., 2003).

However, as astrocytic atrophy in animals submitted to powdered diet seems more severe than that in young HD animals, we suggest that the reduction in the masticatory activity may contribute to slow down the blood flow at the dentate gyrus. In contrast, EE and masticatory activity rehabilitation may improve blood flow; differences were minimized, when comparing HD EE 6M vs. HD/SD/HD EE 6M in both morphological types.

This statement makes more sense when the correlation between the characteristics of immunostain-labeled processes for GFAP and the performances of these groups in the tests of learning and spatial memory in the MWM was made. In these tests, we saw that the old IE animals presented poor performance, regardless of diet regime, while rehabilitated animals, were better than those that suffered a reduction in masticatory activity, in the other conditions. Indeed, the rehabilitated ones became comparable to controls including old animals when they were raised in an EE.

In summary, our data indicate that microanatomical changes may cause learning and spatial memory perturbation, expanding the view that memory formation is not exclusively related to neurons, but also involves glial cells (Azevedo et al., 2009; Kol et al., 2020). No direct correlation related learning and morphology, but suggested different astrocytes profiles. Indeed, astrocytes may contribute to learning and memory as signaling centers, and by giving structural and metabolic support, they regulate synaptic activity and recruit energetic resources for memory consolidation (Zorec et al., 2015; Kol et al., 2020). In particular, it has been demonstrated that hippocampus-based contextual memory process alters the morphology of astrocytes in the dentate gyrus originating a novel cell morphofunctional status, which is different from that of reactive states (Choi et al., 2016).

We then speculate that while the complexity of the astroglial branches at 6M may have neuroprotective properties, the reduction in masticatory activity in combination with aging may suppress astrocyte function and metabolic support, thus compromising homeostasis. In contrast, we suggest that EE associated with the rehabilitation of masticatory activity would supply adequate morphofunctional support, minimizing homeostatic abnormalities associated with dysfunctional mastication.

Conversely, in 18M mice, the complexity of astroglial branches may relate to a distinct functional role, closer to the concept of reactive astrogliosis, especially in groups subjected to altered masticatory activity. Compared to astrocytes of normal CNS, reactive astrocytes change their morphology and function, and this is reflected by the altered genetic expression (Wilhelmsson et al., 2006; Sofroniew, 2009; Verkhratsky et al., 2013). In future investigations, it would be of interest to identify gene expression changes in the astrocyte subtypes, such as AST1 and AST2, to differentiate mechanisms at a molecular scale (Pestana et al., 2020). We propose that the reduced masticatory activity may aggravate the chronic low-grade neuroinflammation of aged mice leading to astrocyte morphological changes. The assumption related to the teeth extraction as a stress inductor (Furuzawa et al., 2014) points in the direction that masticatory dysfunction activates the hypothalamo-hypophysal-adrenal axis in a sustainable way (Snyder et al., 2011; Azuma et al., 2017).

Chewing activity during stress conditions has also been argued to improve hippocampal neurogenesis, synaptic plasticity, and cognitive function by attenuating stress hormone effects and their receptor expression, then reducing hypothalamic–pituitary–adrenal (HPA) axis activity. Therefore, masticatory stimulation may be an effective method to modulate normal feedback mechanism of the HPA axis and prevent stress-induced disorders, especially in older people (Azuma et al., 2017). Thus, we suggest that in the aged groups, with altered masticatory activity (HD/SD and HD/SD/HD), there could be a suppression of the negative feedback on the HPA axis and, consequently, of elevated levels of glucocorticoids. This would be particularly important for hippocampal formation, where morphological changes of astrocytes with increased complexity as part of the adaptive response were detected. The aged and enriched environment rehabilitation group (HD/SD/HD 18M EE) not only exhibited better performance in MWM tests, but also showed a reduction in astroglial branch complexity, as compared to the HD/SD group (deprived of masticatory activity group) raised under the same conditions. This finding reinforced the previous assumption that oral rehabilitation combined with sensory-motor and cognitive stimulation may protect against age-related cognitive decline.

In addition to aging, masticatory activity and environment, as well as sex hormones, interfere with the behavior and/or astrocyte number and morphology in the hippocampus. Indeed, there are multiple indications for sexual dimorphism of astrocytes (Acaz-Fonseca et al., 2016), and gonadal hormones appear to regulate GFAP expression and such astrocyte characteristics. Indeed, aged C57Bl6J female mice have 35% more astrocytes than age-matched males (Mouton et al., 2002), and ovariectomized female mice under estrogenic replacement showed a reduced number of astrocytes in the dentate gyrus when compared to an ovariectomized placebo group (Lei et al., 2003). In addition, the treatment of organotypic cultures with estradiol and testosterone increased astrocytic complexity in the hippocampus and the length of their processes (Del Cerro et al., 1995). Because we have used female mice, we cannot exclude differential effects of hormone concentrations once sexual hormones were not assessed. In the present study, giving the hypothetical estrogen-depleted condition of aged females, we may assume that, at least in part, the morphological alterations of astrocytes can derive from estropause.

Taken together, our findings show, for the first time, that IE and reduced mastication acting together may aggravate age-related cognitive decline and may be associated with differential shrinkage effects on AST1 and AST2 of the molecular layer of the dentate gyrus. In addition, long-term EE applied to the masticatory-deprived group reversed the shrinkage effect associated with a reduced mastication and IE.

## Data Availability Statement

The original contributions generated for this study are included in the article/Supplementary Material. Further inquiries can be directed to the corresponding author.

## Ethics Statement

The animal study was reviewed and approved by Ethics Committee in Research with Experimental Animals (CEPAE) of Biological Sciences Institute from Federal University of Pará (Universidade Federal do Pará – UFPA).

## Author Contributions

FSM, CD, and MS participated in the development and methodological design, collection and treatment of data, and analysis and interpretation of data and writing. FSM, LP, CD, and MS participated in the collection and processing of data. DD, DB, and DA participated in the development and methodological design, supervision, analysis and interpretation of data, and writing. DB critically reviewed the manuscript. All authors contributed to the article and approved the submitted version.

## Conflict of Interest

The authors declare that the research was conducted in the absence of any commercial or financial relationships that could be construed as a potential conflict of interest.

## Supplementary Material

**Supplementary Material 1**

Supplementary Table 1 Learning rate percentage (%) at the 4th testing day for the experimental groups (HD, HD/SD, and HD/SD/HD) at the age of 6 and 18 months in both environments (impoverished and enriched).

Supplementary Table 2 Representation of the mean values and standard error for the complexity of the astrocytes of each phenotype (AST1 and AST2) and *t*-student test (*t*), with respective *p*-values (*p*) for the comparison between them, obtained from the test (HD, HD/SD, or HD/SD/HD), for each experimental group, according to the environment (impoverished and enriched), at the ages of 6 and 18 months (6M and 18M).

Supplementary Table 3 Mean of the total distance traveled (cm) on the 4th test day and standard error for the experimental groups (HD, HD/SD, and HD/SD/HD) at ages 6 and 18 months in both environments (impoverished and enriched).

Supplementary Table 4 Mean of total distance walked in the quadrant opposite the platform (cm) on the 4th test day and standard error for the experimental groups (HD, HD/SD, and HD/SD/HD) at the ages of 6 and 18 months, in both environments (impoverished and enriched).

Supplementary Table 5 Mean of swim speed (cm/s) on the 4th test day and standard error for the experimental groups (HD, HD/SD, and HD/SD/HD) at ages 6 and 18 months in both environments (impoverished and enriched).

Supplementary Table 6 Representation of the values obtained after the Analysis of Variance (ANOVA) – three way with the Tukey post-test (*q*) and *p*-value (*p*) significant for the total distance traveled (cm) at the 4th testing day in the different groups by regime, age and environment.

Supplementary Table 7 Representation of the values obtained after the Analysis of Variance (ANOVA) – three way with the Tukey post-test (*q*) and *p*-value (*p*) significant for the total distance traveled in the quadrant opposite the platform (cm) in the different groups by regime, age and environment.

Supplementary Table 8 Representation of values obtained after Analysis of Variance (ANOVA) – three way with Tukey post-test (*q*) and *p*-value (*p*) significant for the mean swimming velocity (cm/s) at the 4th testing day in the different groups by regime, age and environment.

Supplementary Table 9 Body weight (g) of the animals for each experimental group based on the diet regime (HD, HD/SD, and HD/SD/HD), age (6 or 18 months), in both environments (impoverished and enriched).

Supplementary Table 10 Statistically significant values for the comparisons between the body weight of the animals in each experimental group based on the diet regime (HD, HD/SD, and HD/SD/HD), age (6 or 18 months), in the two environments (impoverished and enriched).

Supplementary Table 11 Pearson’s Linear Correlation between body weight and animal learning rate in the Morris Water Maze test, in each experimental group, based on the diet regime (HD, HD/SD, and HD/SD/HD), age (6 or 18 months), in both environments (impoverished and enriched).

**Supplementary Material 2**

Supplementary Table 1 Data obtained for astrocytes from the discriminant analysis made on morphological variables with multimodal index (MMI) > 0.55 for all experimental groups.

Supplementary Table 2 Representation of the mean values and standard error for the complexity of the astrocytes of each phenotype (AST1 and AST2) and *t*-student test (*t*), with respective *p*-values (*p*) for the comparison between them, obtained from the test (HD, HD/SD, or HD/SD/HD), for each experimental group, according to the environment (impoverished and enriched), at the ages of 6 and 18 months (6M and 18M).

Supplementary Table 3 Representation of values obtained after Analysis of Variance (ANOVA) – one way (Fisher-*F*) and *t*-student test (*t*), with respective *p*-values (*p*) in comparison of the complexity of the branches of the astrocytes (HD, HD/SD, and HD/SD/HD) and age (6 months: 6M and 18 months: 18M), raised in an impoverished environment – IE and enriched – EE.

Supplementary Table 4 Representation of values obtained after Analysis of Variance (ANOVA) – one way (Fisher-*F*) and *t*-student test (*t*), with respective *p*-values (*p*) in the comparison of the complexity of the branches of the astrocytes (HD, HD/SD, and HD/SD/HD) and age (6 months: 6M and 18 months: 18M), raised in an impoverished environment – IE and enriched – EE.
