# Supplementary material for: Sedentary Life and Reduced Mastication Impair Spatial Learning and Memory and Differentially Affect Dentate Gyrus Astrocyte Subtypes in the Aged Mice
Source: Front Neurosci. 2021 Apr 15;15:632216. doi: 10.3389/fnins.2021.632216 (PMC8081835; doi:10.3389/fnins.2021.632216)
Supplement: Supplementary Table 1 — Learning rate percentage (%) at the 4th testing day for the experimental groups (HD, HD/SD, and HD/SD/HD) at the age of 6 and 18 months in both environments (impoverished and enriched). [file Table_1.DOCX]

Table 1: Learning rate percentage (%) at the 4^th^ testing day for the experimental groups (HD, HD/SD and HD/SD/HD) at the age of 6 and 18 months in both environments (impoverished and enriched).

|  | **Learning rate percentage (%)** | | | |
| --- | --- | --- | --- | --- |
| **GROUPS** | **Impoverished Environment** | | **Enriched Environment** | |
|  | **6 months** | **18 months** | **6 months** | **18 months** |
| **HD** | 90.19 ± 2.74 | 45.37 ± 8.78 | 69.67 ± 3.41 | 65.42 ± 6.55 |
| **HD/SD** | 43.84 ± 6.02 | 38.16 ± 8.91 | 46.77 ± 7.76 | 42.63 ± 4.26 |
| **HD/SD/HD** | 78.49 ± 4.43 | 44.25 ± 9.89 | 70.56 ± 4.37 | 61.32 ± 5.64 |

Results are expressed as mean ± standard error. HD: hard diet/pellet food and SD: soft diet/powder food.

Table 2: Representation of the values obtained after the Analysis of Variance (ANOVA) - three way in the Tukey post-test (q) and p-value (p) significant for the learning rate percentage (%) at the 4^th^ testing day in the labyrinth for the several experimental groups.

|  | **Significance values of paired samples for the Learning Rate (%) in the Morris Water Maze** | | | | | | | | | | | |
| --- | --- | --- | --- | --- | --- | --- | --- | --- | --- | --- | --- | --- |
| **GROUPS** | ***HD***  ***IE 6M*** | ***HD/SD***  ***IE 6M*** | ***HD/SD/HD***  ***IE 6M*** | ***HD***  ***IE 18M*** | ***HD/SD***  ***IE 18M*** | ***HD/SD/HD***  ***IE 18M*** | ***HD***  ***EE 6M*** | ***HD/SD***  ***EE 6M*** | ***HD/SD/HD***  ***EE 6M*** | ***HD***  ***EE 18M*** | ***HD/SD***  ***EE 18M*** | ***HD/SD/HD***  ***EE 18M*** |
| ***HD***  ***IE 6M*** | - | q _(8)_ = 7.00  p < 0.0001 | - | q _(8)_ = 4.87  p < 0.0012 | - | - | q _(8)_ = 4.69  p < 0.0016 | - | - | - | - | - |
| ***HD/SD***  ***IE 6M*** | q _(8)_ = 7.00  p < 0.0001 | - | q _(8)_ = 4.63  p < 0.0017 | - | - | - | - | - | - | - | - | - |
| ***HD/SD/HD***  ***IE 6M*** | - | q _(8)_ = 4.63  p < 0.0017 | - | - | - | q _(8)_ = 3.16  p < 0.0134 | - | - | - | - | - | - |
| ***HD***  ***IE 18M*** | q _(8)_ = 4.87  p < 0.0012 | - | - | - | - | - | - | - | - | - | - | - |
| ***HD/SD***  ***IE 18M*** | - | - | - | - | - | - | - | - | - | - | - | - |
| ***HD/SD/HD***  ***IE 18M*** | - | - | q _(8)_ = 3.16  p < 0.0134 | - | - | - | - | - | - | - | - | - |
| ***HD***  ***EE 6M*** | q _(8)_ = 4.69  p < 0.0016 | - | - | - | - | - | - | q _(8)_ = 2.70  p < 0.0269 | - | - | - | - |
| ***HD/SD***  ***EE 6M*** | - | - | - | - | - | - | q _(8)_ = 2.70  p < 0.0269 |  | q _(8)_ = 2.67  p < 0.0283 | - | - | - |
| ***HD/SD/HD***  ***EE 6M*** | - | - | - | - | - | - | - | q _(8)_ = 2.67  p < 0.0283 | - | - | - | - |
| ***HD***  ***EE 18M*** | - | - | - | - | - | - | - | - | - | - | q _(8)_ = 2.92  p < 0.0194 | - |
| ***HD/SD***  ***EE 18M*** | - | - | - | - | - | - | - | - | - | q _(8)_ = 2.92  p < 0.0194 | - | q _(8)_ = 2.64  p < 0.0295 |
| ***HD/SD/HD***  ***EE 18M*** | - | - | - | - | - | - | - | - | - | - | q _(8)_ = 2.64  p < 0.0295 | - |

HD, hard diet/pellet food; SD, soft diet/powder food; IE, impoverished environment; EE, enriched environment; 6M, 6 months of age; 18M, 18 months of age.

Table 3: Mean of the total distance traveled (cm) on the 4^th^ test day and standard error for the experimental groups (HD, HD/SD and HD/SD/HD) at ages 6 and 18 months in both environments (impoverished and enriched).

|  | **Total Distance Traveled (cm)** | | | |
| --- | --- | --- | --- | --- |
| **GROUPS** | **Impoverished Environment** | | **Enriched Environment** | |
|  | **6 months** | **18 months** | **6 months** | **18 months** |
| **HD** | 116.23 ± 16.07 | 366.93 ± 145.16 | 202.48 ± 45.87 | 183.82 ± 52.02 |
| **HD/SD** | 297.21 ± 42.50 | 467.93 ± 79.75 | 240.09 ± 76.04 | 411.17 ± 43.85 |
| **HD/SD/HD** | 98.03 ± 19.55 | 281.14 ± 63.03 | 183.35 ± 44.05 | 211.69 ± 38.47 |

Results are expressed as mean ± standard error. HD: hard diet/pellet food and SD: soft diet/powder food.

Table 4: Mean of total distance walked in the quadrant opposite the platform (cm) on the 4^th^ test day and standard error for the experimental groups (HD, HD/SD and HD/SD/HD) at the ages of 6 and 18 months, in both environments (impoverished and enriched).

|  | **Opposite Quadrant Distance (cm)** | | | |
| --- | --- | --- | --- | --- |
| **GROUPS** | **Impoverished Environment** | | **Enriched Environment** | |
|  | **6 months** | **18 months** | **6 months** | **18 months** |
| **HD** | 19.69 ± 6.54 | 101.45 ± 34.03 | 53.45 ± 19.24 | 51.69 ± 14.62 |
| **HD/SD** | 83.03 ± 18.00 | 107.44 ± 23.77 | 83.90 ± 24.76 | 132.59 ± 25.83 |
| **HD/SD/HD** | 25.43 ± 8.12 | 58.95 ± 24.59 | 51.81 ± 14.07 | 70.08 ± 15.55 |

Results are expressed as mean ± standard error. HD: hard diet/pellet food and SD: soft diet/powder food.

Table 5: Mean of swim speed (cm/s) on the 4^th^ test day and standard error for the experimental groups (HD, HD/SD and HD/SD/HD) at ages 6 and 18 months in both environments (impoverished and enriched).

|  | **Swim Speed (cm/s)** | | | |
| --- | --- | --- | --- | --- |
| **GROUPS** | **Impoverished Environment** | | **Enriched Environment** | |
|  | **6 months** | **18 months** | **6 months** | **18 months** |
| **HD** | 7.35 ± 0.78 | 10.65 ± 1.32 | 8.63 ± 1.33 | 7.36 ± 0.79 |
| **HD/SD** | 9.71 ± 0.63 | 13.09 ± 1.13 | 9.21 ± 0.80 | 11.24 ± 0.72 |
| **HD/SD/HD** | 6.39 ± 0.75 | 9.05 ± 0.83 | 9.03 ± 1.52 | 9.05 ± 0.58 |

Results are expressed as mean ± standard error. HD: hard diet/pellet food and SD: soft diet/powder food.

Table 6: Representation of the values obtained after the Analysis of Variance (ANOVA) - three way with the Tukey post-test (q) and p-value (p) significant for the total distance traveled (cm) at the 4^th^ testing day in the different groups by regime, age and environment.

|  | **Significance values of paired samples for the total distance traveled (cm) in the Morris Aquatic Labyrinth** | | | | | | | | | | | |
| --- | --- | --- | --- | --- | --- | --- | --- | --- | --- | --- | --- | --- |
| **GROUPS** | ***HD***  ***IE 6M*** | ***HD/SD***  ***IE 6M*** | ***HD/SD/HD***  ***IE 6M*** | ***HD***  ***IE 18M*** | ***HD/SD***  ***IE 18M*** | ***HD/SD/HD***  ***IE 18M*** | ***HD***  ***EE 6M*** | ***HD/SD***  ***EE 6M*** | ***HD/SD/HD***  ***EE 6M*** | ***HD***  ***EE 18M*** | ***HD/SD***  ***EE 18M*** | ***HD/SD/HD***  ***EE 18M*** |
| ***HD***  ***IE 6M*** | - | q _(8)_ = 3.98  p < 0.004 | - | - | - | - | - | - | - | - | - | - |
| ***HD/SD***  ***IE 6M*** | q _(8)_ = 3.98  p < 0.004 | - | q _(8)_ = 4.26  p < 0.0028 | - | - | - | - | - | - | - | - | - |
| ***HD/SD/HD***  ***IE 6M*** | - | q _(8)_ = 4.26  p < 0.0028 | - | - | - | q _(8)_ = 2.77  p < 0.024 | - | - | - | - | - | - |
| ***HD***  ***IE 18M*** | - | - | - | - | - | - | - | - | - | - | - | - |
| ***HD/SD***  ***IE 18M*** | - | - | - | - | - | - | - | - | - | - | - | - |
| ***HD/SD/HD***  ***IE 18M*** | - | - | q _(8)_ = 2.77  p < 0.024 | - | - | - | - | - | - | - | - | - |
| ***HD***  ***EE 6M*** | - | - | - | - | - | - | - | - | - | - | - | - |
| ***HD/SD***  ***EE 6M*** | - | - | - | - | - | - | - | - | - | - | - | - |
| ***HD/SD/HD***  ***EE 6M*** | - | - | - | - | - | - | - | - | - | - | - | - |
| ***HD***  ***EE 18M*** | - | - | - | - | - | - | - | - | - | - | q _(8)_ = 3.34  p < 0.010 | - |
| ***HD/SD***  ***EE 18M*** | - | - | - | - | - | - | - | - | - | q _(8)_ = 3.34  p < 0.010 | - | q _(8)_ = 3.42  p < 0.0091 |
| ***HD/SD/HD***  ***EE 18M*** | - | - | - | - | - | - | - | - | - | - | q _(8)_ = 3.42  p < 0.0091 | - |

HD, hard diet/pellet food; SD, soft diet/powder food; IE, impoverished environment; EE, enriched environment; 6M, 6 months of age; 18M, 18 months of age.

Table 7: Representation of the values obtained after the Analysis of Variance (ANOVA) - three way with the Tukey post-test (q) and p-value (p) significant for the total distance traveled in the quadrant opposite the platform (cm) in the different groups by regime, age and environment.

|  | **Significance values of paired samples for the total distance traveled in the Opposite Platform Quadrant (cm) in the Morris Aquatic Labyrinth** | | | | | | | | | | | |
| --- | --- | --- | --- | --- | --- | --- | --- | --- | --- | --- | --- | --- |
| **GROUPS** | ***HD***  ***IE 6M*** | ***HD/SD***  ***IE 6M*** | ***HD/SD/HD***  ***IE 6M*** | ***HD***  ***IE 18M*** | ***HD/SD***  ***IE 18M*** | ***HD/SD/HD***  ***IE 18M*** | ***HD***  ***EE 6M*** | ***HD/SD***  ***EE 6M*** | ***HD/SD/HD***  ***EE 6M*** | ***HD***  ***EE 18M*** | ***HD/SD***  ***EE 18M*** | ***HD/SD/HD***  ***EE 18M*** |
| ***HD***  ***IE 6M*** | - | q _(8)_ = 3.31  p < 0.01 | - | q _(8)_ = 2.36  p < 0.046 | - | - | - | - | - | - | - | - |
| ***HD/SD***  ***IE 6M*** | q _(8)_ = 3.31  p < 0.01 | - | q _(8)_ = 2.92  p < 0.019 | - | - | - | - | - | - | - | - | - |
| ***HD/SD/HD***  ***IE 6M*** | - | q _(8)_ = 2.92  p < 0.019 | - | - | - | - | - | - | - | - | - | - |
| ***HD***  ***IE 18M*** | q _(8)_ = 2.36  p < 0.046 | - | - | - | - | - | - | - | - | - | - | - |
| ***HD/SD***  ***IE 18M*** | - | - | - | - | - | - | - | - | - | - | - | - |
| ***HD/SD/HD***  ***IE 18M*** | - | - | - | - | - | - | - | - | - | - | - | - |
| ***HD***  ***EE 6M*** | - | - | - | - | - | - | - | - | - | - | - | - |
| ***HD/SD***  ***EE 6M*** | - | - | - | - | - | - | - | - | - | - | - | - |
| ***HD/SD/HD***  ***EE 6M*** | - | - | - | - | - | - | - | - | - | - | - | - |
| ***HD***  ***EE 18M*** | - | - | - | - | - | - | - | - | - | - | q _(8)_ = 2.73  p < 0.026 | - |
| ***HD/SD***  ***EE 18M*** | - | - | - | - | - | - | - | - | - | q _(8)_ = 2.73  p < 0.026 | - | - |
| ***HD/SD/HD***  ***EE 18M*** | - | - | - | - | - | - | - | - | - | - | - | - |

HD, hard diet/pellet food; SD, soft diet/powder food; IE, impoverished environment; EE, enriched environment; 6M, 6 months of age; 18M, 18 months of age.

Table 8: Representation of values obtained after Analysis of Variance (ANOVA) - three way with Tukey post-test (q) and p-value (p) significant for the mean swimming velocity (cm/s) at the 4^th^ testing day in the different groups by regime, age and environment.

|  | **Significance values of paired samples for the mean swimming speed (cm/s) in the Morris Aquatic Labyrinth** | | | | | | | | | | | |
| --- | --- | --- | --- | --- | --- | --- | --- | --- | --- | --- | --- | --- |
| **GROUPS** | ***HD***  ***IE 6M*** | ***HD/SD***  ***IE 6M*** | ***HD/SD/HD***  ***IE 6M*** | ***HD***  ***IE 18M*** | ***HD/SD***  ***IE 18M*** | ***HD/SD/HD***  ***IE 18M*** | ***HD***  ***EE 6M*** | ***HD/SD***  ***EE 6M*** | ***HD/SD/HD***  ***EE 6M*** | ***HD***  ***EE 18M*** | ***HD/SD***  ***EE 18M*** | ***HD/SD/HD***  ***EE 18M*** |
| ***HD***  ***IE 6M*** | - | q _(8)_ = 2.35  p < 0.046 | - | - | - | - | - | - | - | - | - | - |
| ***HD/SD***  ***IE 6M*** | q _(8)_ = 2.35  p < 0.046 | - | q _(8)_ = 3.39  p < 0.0095 | - | q _(8)_ = 2.61  p < 0.031 | - | - | - | - | - | - | - |
| ***HD/SD/HD***  ***IE 6M*** | - | q _(8)_ = 3.39  p < 0.0095 | - | - | - | q _(8)_ = 2.39  p < 0.043 | - | - | - | - | - | - |
| ***HD***  ***IE 18M*** | - | - | - | - | - | - | - | - | - | - | - | - |
| ***HD/SD***  ***IE 18M*** | - | q _(8)_ = 2.61  p < 0.031 | - | - | - | q _(8)_ = 2.88  p < 0.02 | - | - | - | - | - | - |
| ***HD/SD/HD***  ***IE 18M*** | - | - | q _(8)_ = 2.39  p < 0.043 |  | q _(8)_ = 2.88  p < 0.02 | - | - | - | - | - | - | - |
| ***HD***  ***EE 6M*** | - | - | - | - | - | - | - | - | - | - | - | - |
| ***HD/SD***  ***EE 6M*** | - | - | - | - | - | - | - | - | - | - | - | - |
| ***HD/SD/HD***  ***EE 6M*** | - | - | - | - | - | - | - | - | - | - | - | - |
| ***HD***  ***EE 18M*** | - | - | - | - | - | - | - | - | - | - | q _(8)_ = 3.62  p < 0.0068 | - |
| ***HD/SD***  ***EE 18M*** | - | - | - | - | - | - | - | - | - | q _(8)_ = 3.62  p < 0.0068 | - | q _(8)_ = 2.35  p < 0.0467 |
| ***HD/SD/HD***  ***EE 18M*** | - | - | - | - | - | - | - | - | - | - | q _(8)_ = 2.35  p < 0.0467 | - |

HD, hard diet/pellet food; SD, soft diet/powder food; IE, impoverished environment; EE, enriched environment; 6M, 6 months of age; 18M, 18 months of age.

Table 9: Body weight (g) of the animals for each experimental group based on the diet regime (HD, HD/SD and HD/SD/HD), age (6 or 18 months), in both environments (impoverished and enriched).

|  | **Body Weight (g)** | | | |
| --- | --- | --- | --- | --- |
| **GROUPS** | **Impoverished Environment** | | **Enriched Environment** | |
|  | **6 months** | **18 months** | **6 months** | **18 months** |
| **HD** | 75.96 ± 3.69 | 75.32 ± 1.45 | 50.94 ± 1.25 | 65.21 ± 4.41 |
| **HD/SD** | 55.04 ± 2.05 | 53.44 ± 3.61 | 44.32 ± 3.31 | 51.21 ± 2.67 |
| **HD/SD/HD** | 71.56 ± 2.93 | 57.65 ± 4.10 | 51.70 ± 5.27 | 54.85 ± 2.09 |

Results are expressed as mean ± standard error. HD: hard diet/pellet food and SD: soft diet/powder food.

Table 10: Statistically significant values for the comparisons between the body weight of the animals in each experimental group based on the diet regime (HD, HD/SD and HD/SD/HD), age (6 or 18 months), in the two environments (impoverished and enriched).

|  | **Statistically Significant Values** | | | |
| --- | --- | --- | --- | --- |
| **COMPARISONS** | **Impoverished Environment** | | **Enriched Environment** | |
|  | **6 months** | **18 months** | **6 months** | **18 months** |
| **ANOVA-*one way*** | F_(2,12)_ = 13.83  p = 0.01 | F_(2,12)_ = 12.63  p = 0.0014 | F_(2,12)_ = 1.23  p = 0.328 | F_(2,12)_ = 5.10  p = 0.025 |
| **HD *vs* HD/SD** | t_(8)_ = 4.99  p < 0.001 | t_(8)_ = 4.74  p < 0.001 | - | t_(8)_ = 3.08  p = 0.0095 |
| **HD *vs* HD/SD/HD** | t_(8)_ = 1.05  p > 0.05 | t_(8)_ = 3.82  p = 0.0024 | - | t_(8)_ = 2.28  p < 0.0418 |
| **HD/SD *vs* HD/SD/HD** | t_(8)_ = 3.94  p = 0.002 | t_(8)_ = 0.91  p > 0.05 | - | t_(8)_ = 0.80  p > 0.05 |

HD: hard diet/pellet food and SD: soft diet/powder.

Table 11: Pearson’s Linear Correlation between body weight and animal learning rate in the Morris Water Maze test, in each experimental group, based on the diet regime (HD, HD/SD and HD/SD/HD), age (6 or 18 months), in both environments (impoverished and enriched).

|  | **Pearson's Linear Correlation** | | | |
| --- | --- | --- | --- | --- |
| **COMPARISONS** | **Impoverished Environment** | | **Enriched Environment** | |
|  | **6 months** | **18 months** | **6 months** | **18 months** |
| **HD** | r = 0.76  R^2^ = 0.57  p = 0.138 | r = - 0.50  R^2^ = 0.25  p = 0.387 | r = - 0.49  R^2^ = 0.24  p = 0.397 | r = 0.70  R^2^ = 0.48  p = 0.19 |
| **HD/SD** | r = - 0.89  R^2^ = 0.79  p = 0.0443 | r = 0.16  R^2^ = 0.02  p = 0.80 | r = 0.06  R^2^ = 0.004  p = 0.92 | r = 0.49  R^2^ = 0.24  p = 0.40 |
| **HD/SD/HD** | r = -0.75  R^2^ = 0.56  p = 0.146 | r = - 0.32  R^2^ = 0.10  p = 0.60 | r = - 0.28  R^2^ = 0.08  p = 0.65 | r = 0.44  R^2^ = 0.19  p = 0.46 |

HD: hard diet/pellet food and SD: soft diet/powder.
