# Supplementary material for: Sedentary Life and Reduced Mastication Impair Spatial Learning and Memory and Differentially Affect Dentate Gyrus Astrocyte Subtypes in the Aged Mice
Source: Front Neurosci. 2021 Apr 15;15:632216. doi: 10.3389/fnins.2021.632216 (PMC8081835; doi:10.3389/fnins.2021.632216)
Supplement: Supplementary Table 1 — Data obtained for astrocytes from the discriminant analysis made on morphological variables with multimodal index (MMI) > 0.55 for all experimental groups. [file Table_2.DOCX]

Table 1: Data obtained for astrocytes from the discriminant analysis made on morphological variables with multimodal index (MMI)> 0.55 for all experimental groups.

|  | **Wilks’ Lambda** | **Partial Lambda** | **F-remove** | **p-level** | **Toler.** | **1-Toler. (R-Sqr.)** |
| --- | --- | --- | --- | --- | --- | --- |
| **HD IE 6M** | | | | | | |
| Total Branches Volume | 0.306286 | 0.997065 | 0.4268 | 0.514602 | 0.689593 | 0.310407 |
| Convexity of soma | 0.309650 | 0.986236 | 2.0237 | 0.157013 | 0.948610 | 0.051390 |
| Solidity of soma | 0.305395 | 0.999976 | 0.0035 | 0.952730 | 0.959636 | 0.040364 |
| **Complexity** | 0.646897 | 0.472080 | 162.1511 | 0.000000 | 0.683340 | 0.316660 |
| **HD/SD IE 6M** | | | | | | |
| Solidity of soma | 0.401628 | 0.989939 | 1.4940 | 0.223547 | 0.994214 | 0.005786 |
| **Complexity** | 0.995693 | 0.399307 | 221.1380 | 0.000000 | 0.994214 | 0.005786 |
| **HD/SD/HD IE 6M** | | | | | | |
| Tortuosity | 0.390503 | 0.998292 | 0.2480 | 0.619210 | 0.789471 | 0.210529 |
| Convexity of soma | 0.394847 | 0.987309 | 1.8638 | 0.174298 | 0.808723 | 0.191277 |
| **Complexity** | 0.775535 | 0.502667 | 143.4614 | 0.000000 | 0.918395 | 0.081605 |
| **Convex Hull – Volume** | 0.488752 | 0.797615 | 36.7920 | 0.000000 | 0.965940 | 0.034060 |
| **HD IE 18M** | | | | | | |
| **Convexity of soma** | 0.996061 | 0.284004 | 370.5977 | 0.000000 | 0.878934 | 0.121066 |
| **Solidity of soma** | 0.316473 | 0.893871 | 17.4533 | 0.000050 | 0.878934 | 0.121066 |
| **HD/SD IE 18M** | | | | | | |
| Aspect ratio of soma | 0.301077 | 0.999911 | 0.0129 | 0.909747 | 0.180126 | 0.819875 |
| Convexity of soma | 0.306559 | 0.982032 | 2.6530 | 0.105525 | 0.221843 | 0.778157 |
| Shape factor of soma | 0.302388 | 0.995577 | 0.6442 | 0.423524 | 0.098807 | 0.901193 |
| **Complexity** | 0.985465 | 0.305491 | 329.6459 | 0.000000 | 0.955817 | 0.044183 |
| **HD/SD/HD IE 18M** | | | | | | |
| Convexity of soma | 0.282341 | 0.993485 | 0.9377 | 0.334496 | 0.419850 | 0.580150 |
| Shape factor of soma | 0.280942 | 0.998433 | 0.2244 | 0.636439 | 0.296889 | 0.703111 |
| Solidity of soma | 0.281040 | 0.998084 | 0.2745 | 0.601170 | 0.579987 | 0.420013 |
| **Complexity** | 0.528893 | 0.530356 | 126.6301 | 0.000000 | 0.609663 | 0.390337 |
| Vertex Analysis (Vc) | 0.280930 | 0.998476 | 0.2183 | 0.641086 | 0.633828 | 0.366172 |
| **Convex Hull – Volume** | 0.336415 | 0.833796 | 28.5047 | 0.000000 | 0.931521 | 0.068479 |
| **HD EE 6M** | | | | | | |
| Convexity of soma | 0.462079 | 0.995908 | 0.5999 | 0.439869 | 0.974814 | 0.025186 |
| Solidity of soma | 0.461290 | 0.997610 | 0.3498 | 0.555153 | 0.981538 | 0.018462 |
| **Complexity** | 0.993084 | 0.463393 | 169.0676 | 0.000000 | 0.993061 | 0.006939 |
| **HD/SD EE 6M** | | | | | | |
| Aspect ratio of soma | 0.408232 | 0.972766 | 4.0034 | 0.047300 | 0.082600 | 0.917400 |
| Convexity of soma | 0.411011 | 0.966190 | 5.0041 | 0.026835 | 0.067682 | 0.932318 |
| Shape factor of soma | 0.408800 | 0.971414 | 4.2081 | 0.042057 | 0.029533 | 0.970467 |
| Solidity of soma | 0.402701 | 0.986127 | 2.0117 | 0.158263 | 0.257630 | 0.742370 |
| **Complexity** | 0.856034 | 0.463900 | 165.2562 | 0.000000 | 0.984905 | 0.015095 |
| **Convex Hull – Volume** | 0.416131 | 0.954301 | 6.8478 | 0.009829 | 0.974697 | 0.025303 |
| **HD/SD/HD EE 6M** | | | | | | |
| Convexity of soma | 0.399498 | 0.999786 | 0.0311 | 0.860349 | 0.989044 | 0.010957 |
| Solidity of soma | 0.399444 | 0.999920 | 0.0117 | 0.914132 | 0.983275 | 0.016725 |
| **Complexity** | 0.856344 | 0.466416 | 165.8815 | 0.000000 | 0.948749 | 0.051251 |
| **Convex Hull – Volume** | 0.473749 | 0.843089 | 26.9867 | 0.000001 | 0.954608 | 0.045392 |
| **HD EE 18M** | | | | | | |
| Convexity of soma | 0.407958 | 0.995772 | 0.6072 | 0.437147 | 0.865832 | 0.134168 |
| Solidity of soma | 0.408551 | 0.994328 | 0.8157 | 0.367946 | 0.873199 | 0.126801 |
| Segments/mm | 0.413233 | 0.983061 | 2.4640 | 0.118695 | 0.953752 | 0.046248 |
| **Complexity** | 0.846824 | 0.479714 | 155.0943 | 0.000000 | 0.960216 | 0.039784 |
| **Convex Hull – Volume** | 0.455798 | 0.891258 | 17.4475 | 0.000051 | 0.978038 | 0.021962 |
| **HD/SD EE 18M** | | | | | | |
| Convexity of soma | 0.383241 | 0.991237 | 1.2818 | 0.259425 | 0.878435 | 0.121566 |
| Solidity of soma | 0.380149 | 0.999299 | 0.1017 | 0.750209 | 0.880986 | 0.119014 |
| **Complexity** | 0.958828 | 0.396195 | 220.9813 | 0.000000 | 0.881325 | 0.118675 |
| Convex Hull – Volume | 0.388738 | 0.977221 | 3.3800 | 0.068039 | 0.896921 | 0.103079 |
| **HD/SD/HD EE 18M** | | | | | | |
| Aspect ratio of soma | 0.367486 | 0.999672 | 0.0469 | 0.828925 | 0.974520 | 0.025481 |
| Convexity of soma | 0.367405 | 0.999891 | 0.0155 | 0.901001 | 0.965929 | 0.034071 |
| Solidity of soma | 0.367833 | 0.998729 | 0.1819 | 0.670351 | 0.958822 | 0.041178 |
| **Complexity** | 0.921044 | 0.398857 | 215.5243 | 0.000000 | 0.988252 | 0.011748 |
| Convex Hull – Volume | 0.373696 | 0.983058 | 2.4645 | 0.118657 | 0.988660 | 0.011340 |

Representation of all morphological variables with multimodality index> 0.55 (left of the Table), for all experimental groups, according to the diet regime (HD, HD/SD or HD/SD/HD), at age 6 or 18 months (6M or 18M) and environment (impoverished - IE or enriched - EE). The highlighted variables (in bold) are those with significant discriminant analysis, that is, which contributed most to the set of observed morphological variations. Note that complexity is the significant morphological variable in all experimental groups, except on the IE 18M HD, being the one chosen for comparison between groups. Data obtained in the discriminant analysis are presented in the rest of the table. HD: hard diet/pellet food and SD: soft diet/powder food.

Table 2: Representation of the mean values and standard error for the complexity of the astrocytes of each phenotype (AST1 and AST2) and t-student test (t), with respective p-values (p) for the comparison between them, obtained from the test (HD, HD/SD or HD/SD/HD), for each experimental group, according to the environment (impoverished and enriched), at the ages of 6 and 18 months (6M and 18M).

|  | **Complexity of Astrocytes AST1 and AST2: mean ± standard error and statistical significance values for paired samples** | | | | | |
| --- | --- | --- | --- | --- | --- | --- |
|  | **Impoverished Environment** | | | **Enriched Environment** | | |
| **GROUPS** | **AST1** | **AST2** | ***t-*student test** | **AST1** | **AST2** | ***t-*student test** |
| ***HD – 6M*** | 20,368.17 ± 1,107.49 | 5,426.33 ± 308.83 | t_(36)_ = 13  p < 0.0001 | 17,363.70 ± 1,963.07 | 4,250.85 ± 224.97 | t_(25)_ = 6.64  p < 0.0001 |
| ***HD/SD – 6M*** | 12,359.72 ± 999.59 | 3,588.68 ± 165.27 | t_(30)_ = 8.66  p < 0.0001 | 13,690.80 ± 956.83 | 3,385.73 ± 185.71 | t_(55)_ = 10.57  p < 0.0001 |
| ***HD/SD/HD – 6M*** | 13,409.92 ± 1,220.67 | 3,598.57 ± 187.67 | t_(42)_ = 7.94  p < 0.0001 | 18,600.17 ± 2,029.79 | 4,312.93 ± 241.70 | t_(30)_ = -6.99  p < 0.0001 |
| ***HD – 18M*** | 5,788.87 ± 550.59 | 5,240.66 ± 496.26 | t_(109)_ = -0.74  p = 0.46 | 7,881.95 ± 431.46 | 2,508.11 ± 130.25 | t_(78)_ = -11.92  p < 0.0001 |
| ***HD/SD – 18M*** | 16,740.59 ± 942.84 | 4,672.49 ± 237.15 | t_(29)_ = 12.40  p < 0.0001 | 34,274.84 ± 3,704.92 | 7,014.61 ± 405.90 | t_(22)_ = 7.31  p < 0.0001 |
| ***HD/SD/HD – 18M*** | 20,363.20 ± 1,910.34 | 4,280.63 ± 235.37 | t_(22)_ = 8.36  p < 0.0001 | 15,210.25 ± 1,250.74 | 3,688.81 ± 181.21 | t_(32)_ = 9.12  p < 0.0001 |

HD: hard diet/pellet food and SD: soft diet/powder food.

Table 3: Representation of values obtained after Analysis of Variance (ANOVA) - one way (Fisher-F) and *t*-student test (t), with respective p-values (p) in comparison of the complexity of the branches of the astrocytes (HD, HD/SD and HD/SD/HD) and age (6 months: 6M and 18 months: 18M), raised in an impoverished environment - IE and enriched - EE.

|  | **Statistically Significant Values for Astrocytes AST1 Complexity** | | | | | | |
| --- | --- | --- | --- | --- | --- | --- | --- |
| **VARIABLES** | **ANOVA**  ***one way*** | **HD *vs* HD** | **HD *vs* HD/SD** | **HD *vs* HD/SD/HD** | **HD/SD *vs* HD/SD** | **HD/SD *vs* HD/SD/HD** | **HD/SD/HD *vs* HD/SD/HD** |
| **IE 6M** | F_(2,99)_ = 13.50  p < 0.0001 | - | t_(59)_ = 4.65  p < 0.001 | t_(71)_ = 4.39  p < 0.001 | - | t_(68)_ = 0.64  p > 0.05 | - |
| **IE 18M** | F_(2,89)_ = 58.90  p < 0.0001 | - | t_(68)_ = 7.87  p < 0.001 | t_(63)_ = 9.81  p < 0.001 | - | t_(47)_ = 2.23  p = 0.0285 | - |
| **EE 6M** | F_(2,104)_ = 3.29  p = 0.04 | - | t_(75)_ = 1.69  p > 0.05 | t_(53)_ = 0.51  p > 0.05 | - | t_(80)_ = 2.39  p = 0.0184 | - |
| **EE 18M** | F_(2,118)_ = 78.19  p < 0.0001 | - | t_(87)_ = 12.50  p < 0.001 | t_(97)_ = 3.97  p < 0.001 | - | t_(52)_ = 8.00  p < 0.001 | - |
| **IE 6M vs IE 18M** | - | t_(46)_ = 11.79  p < 0.0001 | - | - | t_(54)_ = -3.18  p = 0.0024 | - | t_(61)_ = -3.19  p = 0.0022 |
| **EE 6M vs EE 18M** | - | t_(26)_ = 4.72  p < 0.0001 | - | - | t_(24)_ = -5.38  p < 0.0001 | - | t_(49)_ = 1.42  p = 0.16 |
| **IE 6M vs EE 6M** | - | t_(39)_ = 1.33  p = 0.19 | - | - | t_(79)_ = -0.90  p = 0.37 | - | t_(69)_ = -2.31  p = 0.024 |
| **IE 18M vs EE 18M** | - | t_(108)_ = -3.01  p = 0.003 | - | - | t_(31)_ = -3.34  p = 0.002 | - | t_(52)_ = 2.36  p = 0.022 |

HD: hard diet/pellet food and SD: soft diet/powder food.

Table 4: Representation of values obtained after Analysis of Variance (ANOVA) - one way (Fisher-F) and t-student test (t), with respective p-values (p) in the comparison of the complexity of the branches of the astrocytes (HD, HD/SD and HD/SD/HD) and age (6 months: 6M and 18 months: 18M), raised in an impoverished environment - IE and enriched - EE .

|  | **Statistically Significant Values for Astrocytes AST2 Complexity** | | | | | | |
| --- | --- | --- | --- | --- | --- | --- | --- |
| **VARIABLES** | **ANOVA**  ***one way*** | **HD *vs* HD** | **HD *vs* HD/SD** | **HD *vs* HD/SD/HD** | **HD/SD *vs* HD/SD** | **HD/SD *vs* HD/SD/HD** | **HD/SD/HD *vs* HD/SD/HD** |
| **IE 6M** | F_(2,345)_ = 21.24  p < 0.0001 | - | t_(237)_ = 5.72  p < 0.01 | t_(225)_ = 5.53  p < 0.01 | - | t_(228)_ = 0.03  p > 0.05 | - |
| **IE 18M** | F_(2,355)_ = 2.11  p = 0.12 | - | - | - | - | - | - |
| **EE 6M** | F_(2,340)_ = 4.92  p = 0.008 | - | t_(221)_ = 2.68  p < 0.01 | t_(243)_ = 0.203  p > 0.05 | - | t_(216)_ = 2.85  p < 0.01 | - |
| **EE 18M** | F_(2,324)_ = 60.03  p < 0.0001 | - | t_(208)_ = 10.08  p < 0.01 | t_(197)_ = 2.59  p < 0.01 | - | t_(243)_ = 8.23  p < 0.01 | - |
| **IE 6M vs IE 18M** | - | t_(180)_ = 0.32  p = 0.75 | - | - | t_(217)_ = -3.75  p = 0.0002 | - | t_(230)_ = -2.27  p = 0.024 |
| **EE 6M vs EE 18M** | - | t_(189)_ = 6.70  p < 0.0001 | - | - | t_(176)_ = -8.13  p < 0.0001 | - | t_(219)_ = 2.07  p = 0.04 |
| **IE 6M vs EE 6M** | - | t_(217)_ = 3.08  p = 0.0024 | - | - | t_(217)_ = 0.81  p = 0.42 | - | t_(218)_ = -2.33  p = 0.02 |
| **IE 18M vs EE 18M** | - | t_(120)_ = 5.33  p < 0.0001 | - | - | t_(204)_ = -4.98  p < 0.0001 | - | t_(233)_ = 1.99  p = 0.048 |

HD: hard diet/pellet food and SD: soft diet/powder food.
